# Mineral derivatives in alleviating oral mucositis during cancer therapy: a systematic review

**DOI:** 10.7717/peerj.765

**Published:** 2015-02-12

**Authors:** Sonia Lee

**Affiliations:** The Sydney School of Public Health, Edward Ford Building, The University of Sydney, NSW, Australia

**Keywords:** Minerals, Electrolytes, Chemotherapy, Radiotherapy, Neoplasm, Adverse event, Systematic review, Meta-analysis, Cost-effective, Decision analysis

## Abstract

**Objectives.** Oral mucositis (mouth ulcers) is a cancer therapy side effect. Costly treatment interventions are often neglected in favor of cost-effective agents. This review assessed the general efficacy of mineral derivatives (a cost-effective agent) in alleviating oral mucositis (OM) during cancer therapy compared to the standard care, or placebo—including a decision tree to aide healthcare workers.

**Data Sources.** Electronic searches of MEDLINE via OVID, EMBASE, CENTRAL, CANCERLIT via PubMed, and CINAHL via EBSCO (year 2000 to 11 September 2014) were undertaken for randomised controlled trials. A meta-search strategy extracted content from aggregate online databases.

**Review Methods.** Randomized controlled trials were assessed (participants, intervention, outcome, results, and risk of bias) for inclusion. The author abstracted binary and continuous data synthesised to Hedges’ *g* in a random effects model. The primary outcome measures were severity (incidence of peak oral mucositis, duration of oral mucositis, and time to onset); secondary outcome measures were the incidence of pain, and analgesic use. Serum mineral levels, total parenteral nutrition, and adverse events were discussed. The decision tree was mapped using sensitivity, specificity, pre-test and post-test Bayesian probability.

**Results.** 1027 citations were identified and 16 studies were included (*n* = 1120; mean age 49 years). Cancer therapies consisted of chemotherapy, radiotherapy, chemo-radiotherapy, or hematopoietic stem cell transplantation. Outcome mineral derivatives were zinc (*n* = 549), calcium phosphate (*n* = 227), povidone-iodine (*n* = 228), or selenium (*n* = 116). Severity was measured across variable OM grading systems: In 13 studies, individuals in treatment groups (*n* = 958) experienced peak OM less than controls (*g* = −0.47, 95% CI −0.7 to −0.2, *p* = 0.0006); time to OM onset was significantly delayed in treatment than controls (*g* = −0.51, 95% CI−0.8 to −0.2, *p* = 0.0002; five studies); OM mean duration, pain incidences, or analgesics use was not significant. The decision analysis favored selenium.

**Conclusion.** The general positive effect trend suggests individuals taking mineral derivatives during cancer therapies are less likely to experience peak OM than those without. However, significant bias and heterogeneity indicates the need for developing further methods in account of diverse protocols and include novel recordings (serum mineral levels and cell signals) in estimating a uniform true effect.

## Introduction

Oral mucositis is a severe common side effect in over 40% of cancer patients undergoing chemo-radiotherapy (Alessandro Scardina, Pisano & Messina, 2010). In alleviating oral pain, a common practice among physicians is to temporarily halt cancer treatments during chemotherapy or prescribe prophylactic agents during radiotherapy. Targeting oral mucositis, like many cancer therapies, involves a plethora of subjective and objective observations: physical exams, serum and toxicity discrepancies, qualitative feedback, invasive versus non-invasive routes, infection, and oral hygiene (Quinn et al., 2008). Worthington et al. (2013) conducted a large-scale comprehensive review in treating chemo-induced oral mucositis and concluded the best interventions to be cryotherapy (ice chips) and keratinocyte growth factor. This was also recommended by MASCC/ISOO (2014), along with other oral-based or costly treatment options. However, oral-based options could be regarded as analogous to freezing off freckles and stimulating collagen at a beauty spa. It decreases pain and inflammation but may do little in prevention and treatment beyond the superficial dermal.

Assessing pain can be subjective and a complex benchmark for curative purposes (McQuay, Kalso, & Moore , 2010). For the cancer patient, quality of life and cancer therapies are disrupted when severe inflammation causes marked pain (Zylicz, 2013). This inflammation commonly occurs in the mouth and joints from chemo-radiotherapy induced toxicities. In preventing major joint inflammation, magnesium sulfate is optionally administered intravenously once or twice in two hourly drips during chemo-radiotherapy cycles (Kumar, 2012). Magnesium is an essential trace mineral used in emergencies by the World Health Organization in treatments and for maintaining electrolyte balances (Food and Agriculture Organization of the United Nations, World Health Organization , 2004). When magnesium sulfate is not administered intravenously during aggressive chemotherapy, adverse events such as arthritis and oral mucositis are reported higher (Kumar, 2012). It is up to the oncologist to decide whether magnesium sulfate should be administered on the day of treatment cycle (Gaby, 2009). However, earlier time delays from adjusting a patient’s port or radiology bookings may often hinder the use of optionally administering magnesium sulfate. On the other hand, alternative electrolytes are marketed through coconut water and energy drinks. Its efficacy in treating oral mucositis also remains unclear (Helm, 2010; Schang, 2013). Hence, in PICO, this review assessed the effects of the intervention—any mineral derivatives in treating oral mucositis, compared to the standard care or placebo, in any cancer types of all ages, undergoing recent (year 2000 onwards; last 15 years) cancer therapy combinations.

## Methods

### Search strategy

Only randomized controlled trials (RCTs) were included in the review from search databases: MEDLINE via OVID, EMBASE, CENTRAL, CANCERLIT via PubMed, and CINAHL via EBSCO (2000 to 11 September 2014; see ‘ Appendix A ’ for search strategies) based on PRISMA (Preferred Reporting Items for Systematic reviews and Meta-Analyses) and Cochrane guidelines (Higgins, Green & Cochrane Collaboration, 2008). The time frame loosely coincides with recent chemo-radiotherapy combinations from the year 2000 onwards e.g., 5-FU, cisplatin and radiotherapy—neoadjuvant chemoradiation; ECF. Trace minerals regarded essential by the World Health Organization guidelines (Food and Agriculture Organization of the United Nations, World Health Organization , 2004) were combined using Boolean filtered MeSH terms and text words “random,” “control,” and “cancer.” Authors were contacted if full text articles or Supplemental Information were missing. A meta-search strategy was applied to the World Wide Web for articles (conference proceedings, clinical practice guidelines, clinical trials, and pre-prints) not indexed in databases. There was no language restriction on trial eligibility.

### Study selection

The articles underwent a preliminary extraction using a template (Appendix B), and pre-set criteria on study inclusion and exclusion (Table 1). Mineral derivatives were administered as oral rinse, tablet, or intravenously. Trials using other interventions administered concurrently (e.g., vitamins) were excluded. All ages, cancer types, staging, and cancer therapy combinations were included. Significantly biased studies were excluded in the analysis based on a bias appraisal of the strengths and weaknesses in selection and protocol.

**Table 1:** Inclusion and exclusion criteria applied to searched studies.

Inclusion criteria	Exclusion criteria
• Randomized controlled trial	• Exact duplicates from different databases
• Oral complications	• No outcome data
• Mineral derivative as intervention	• No trial
	• Review
	• Vitamins
	• Animal studies
	• Non cancer related
	• Non randomized
	• Supplemental Information

### Quality appraisal

Studies underwent a bias appraisal in accordance with Cochrane Collaboration guidelines using templates in *Revman version 5.2* (Review Manager, 2014). Possible risks (selection, performance, detection, attrition, selective reporting, and other bias) were rated high, low, or unclear by the author and collated into a summary and graph. ‘Selective reporting’ assessed omitted information, and ‘other bias’ for causal analysis errors.

### Outcome measures

The primary outcomes compared severity in independent variables: peak incidence, duration, and onset of oral mucositis (OM) between mineral derivative and control groups (standard care, or placebo) based on severity measures defined by the OM grading systems. The secondary outcomes were the incidence of pain (visual analog scale), and use of analgesics. Serum levels, total parenteral nutrition, and adverse events were discussed.

### Statistical analysis

Dichotomous data was abstracted using a 2 × 2 contingency table for three independent conditions (participants with OM or not; pain or not; analgesics use or not), and continuous data abstracted by mean days, standard deviation, and sample sizes. Studies with no data for an outcome (e.g., some studies did not record analgesics use) were excluded. Studies with missing data and only *p*-values were converted into standardized mean differences using the *Practical Meta-analysis Effect Size Calculator*(online) (Wilson, 2014), and missing standard deviations imputed with similar data (Appendix C) (Wolter, 2007). All data was then pooled by outcome: peak OM incidence, OM duration, time to OM onset, pain incidence, analgesic use, and arbitrarily assigned a 95% confidence interval (CI) and expressed as Hedges’ adjusted *g*—decided a priori in correcting small sample biases and standardizing diverse protocols. A random effects model was decided a priori in account of all effect sizes via a summary estimate of mean distributions, and in minimizing random sampling errors between small and large studies when combining within and between study variances. All outcomes were expressed using the *Comprehensive Metaanalysis version 2.0* (Borenstein et al., 2005). Heterogeneity was assessed using Cochran’s *Q* statistic with *I*^2^ statistic above 50% indicating significant heterogeneity. Publication bias was assessed with Funnel Plots and Egger’s regression model (two-tailed; *p*-value less than 0.05 indicating significant bias) (Rothstein, Sutton & Borenstein, 2005).

### Decision analysis

High quality trials were mapped to a decision tree using *TreeAge Pro 2014 version R2.2* (TreeAge Pro , 2014). The risk of developing peak OM data was imputed by sensitivity, specificity, and pre-test or post-test Bayesian probability (Gelman, 2004). In the first node, equal weight (0.5) was assigned to treatment and controls for each mineral derivative arm. Sensitivity and specificity was calculated using *MedCalc* (Charlie’s Clinical Calculators, 2014) in conjunction with statistical program R (R Core Team, 2014) for subsequent nodes (2 × 2 contingency tables; participants with peak OM or not), and terminal nodes assigned pre-test (control), or post-test (treatment) Bayesian probability which defined ‘risk’ as the pre- or posttest predictive value in developing peak OM (Petitti, 2000). The pre-test (prevalence) of developing OM was set high “0.8” based on high risk groups developing OM during cancer therapy (Sonis, 2009). The tree was then “rolled back” in estimate of probable risks in developing peak OM or not, and in deciding which treatment was the most effective.

## Results

**Figure 1:**
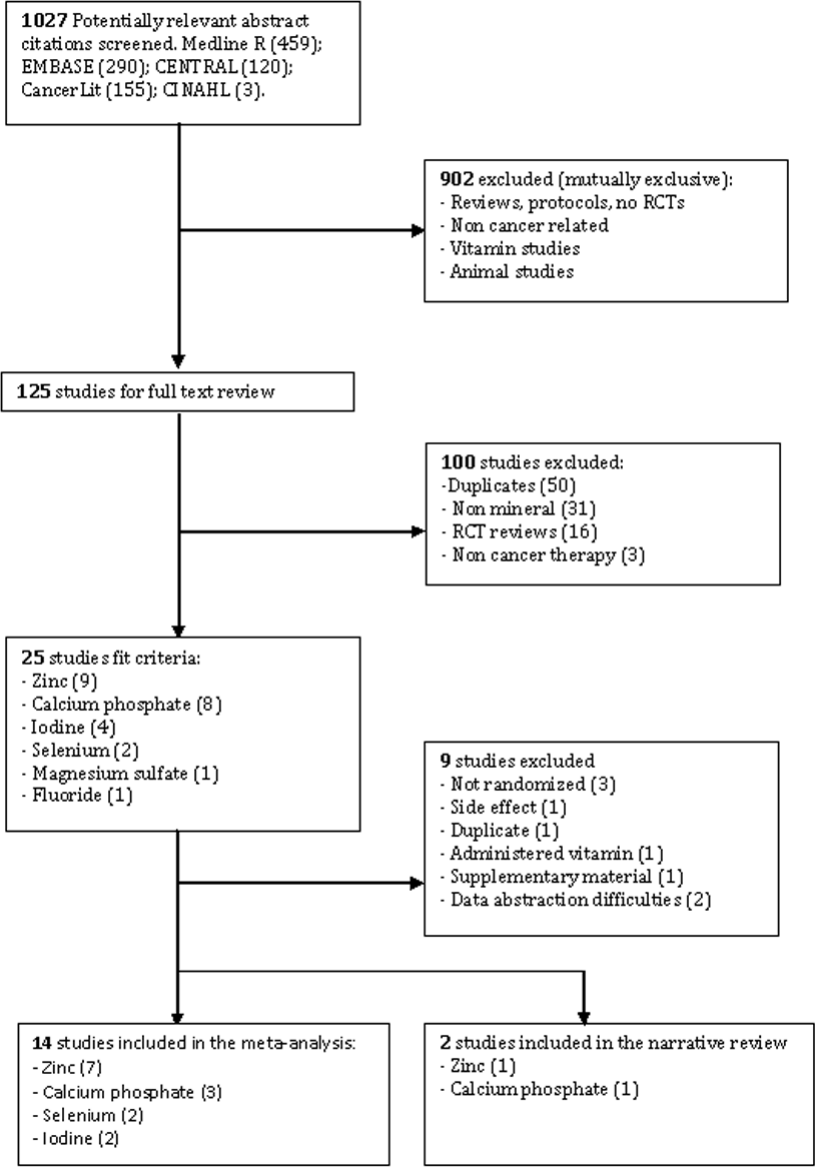
A flow diagram of search results based on the PRISMA statement (Moher et al., 2009).

The search strategy identified 1027 publications screened for inclusion. Sixteen randomized controlled trials met the inclusion criteria (Fig. 1). The meta-search produced studies with insufficient data, and consisted of conference summaries, announcements, policy documents, and Supplemental Information. It was difficult to authenticate and required algorithm tools beyond the scope of this review, hence excluded. Nine studies were also excluded: oral complication listed as side effect (one), study not randomized (three), duplicate publication (one), data on serum levels (two), Supplemental Information on an existing trial (one), and vitamin K1 administered concurrently (one) (see Appendix D, Table 2).

### Quality assessment: selection and measurement risks

**Figure 2:**
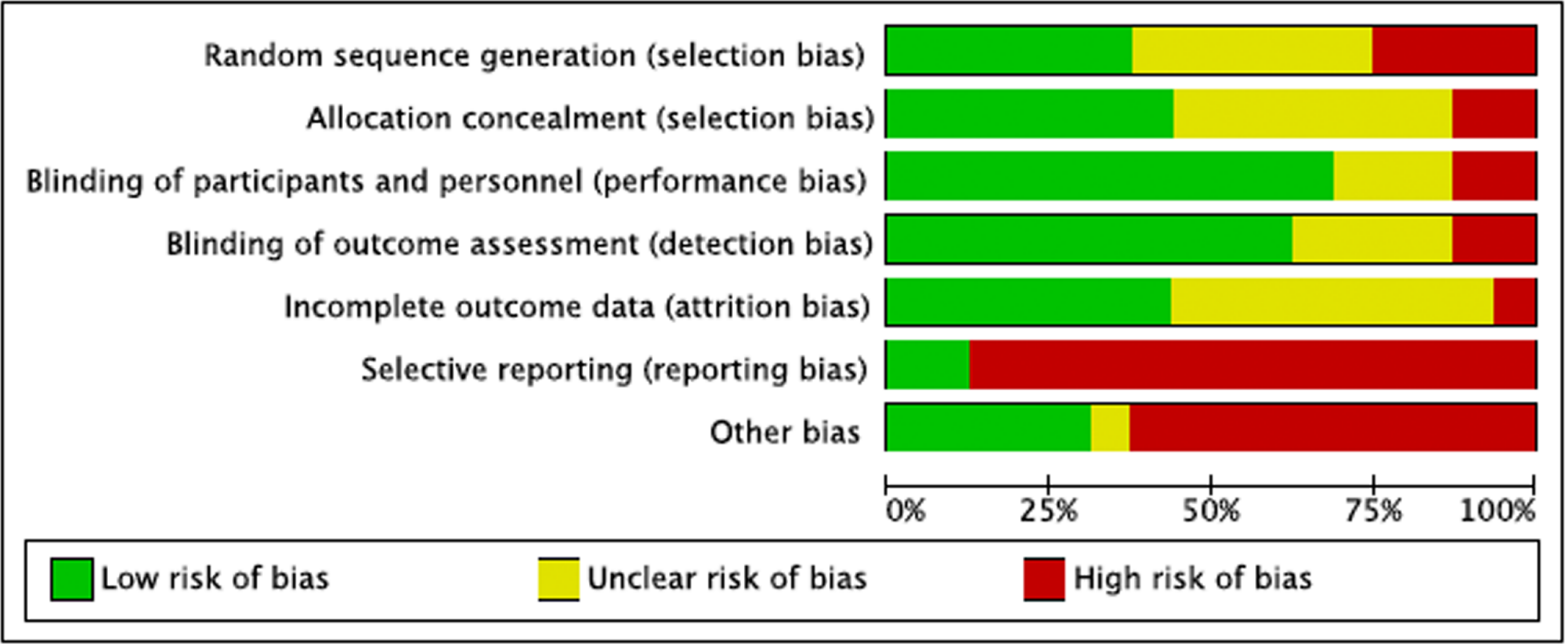
Summary of risks (bias appraisal). A summary of risks across studies based on author rating: low, unclear, or high risk of bias (see Appendix D for details).

**Figure 3:**
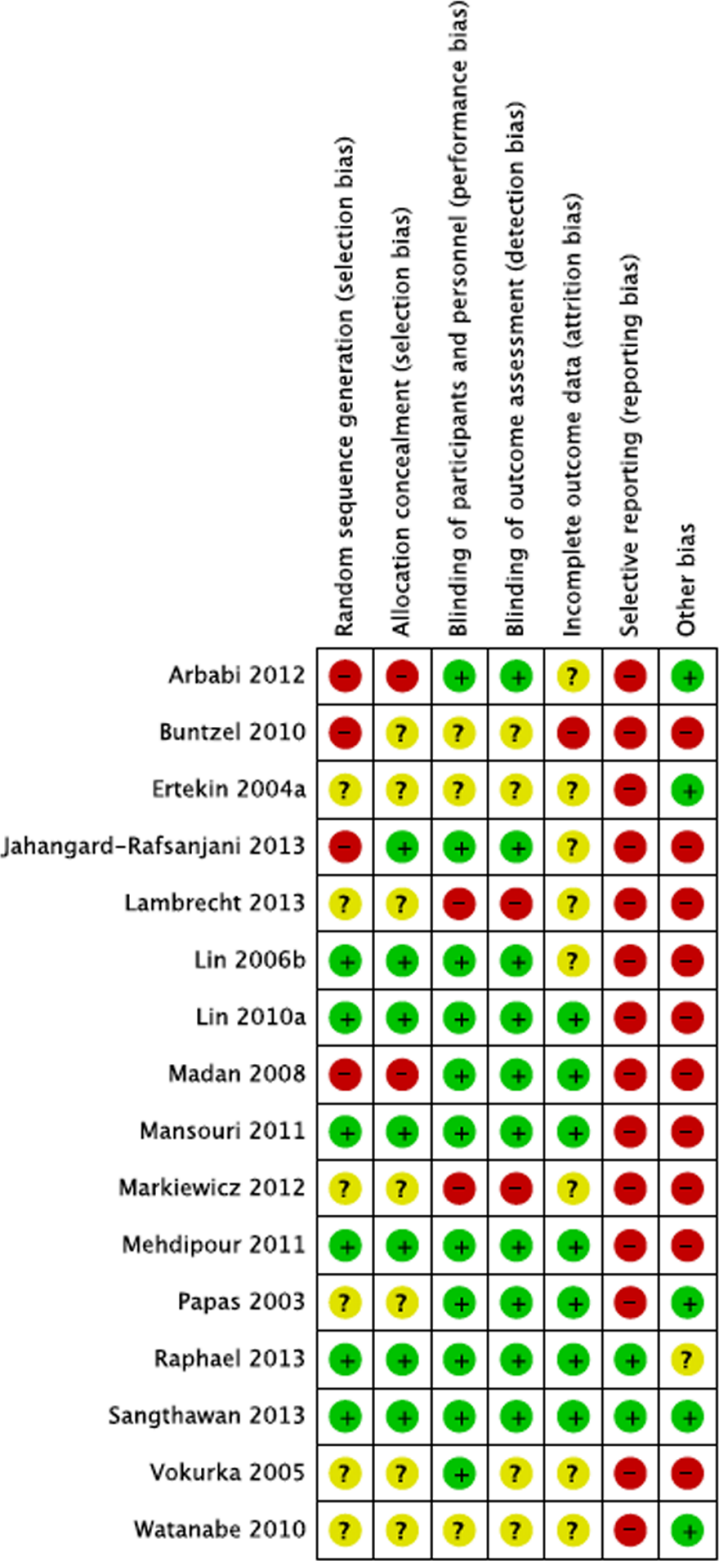
Summary of risk ratings. Detailed risk rating for each study. The “+” sign denotes low risk of bias, “?” unclear risk of bias and “−” clear risk of bias (see Appendix D for details).

Random sequence generation was reported in six out of 16 studies via a computerized method, and allocation concealment in seven studies. Randomized methods were unstated or unclear in the remaining studies. Ten studies explicitly described double-blind methods, one study mentioned “double-blind” but did not specify details (Papas et al., 2003) and one study described blinding as impracticable given the different appearances in treatment and control agents (Markiewicz et al., 2012). Participant completion rates were high in 11 studies that reported attrition rates. Drop out reasons included death (*n* = 11); in one study, one in ten died during treatment with no follow-up (Madan et al., 2008). Treatment refusal or study withdrawal (*n* = 11), opting for herbal and alternative treatments (*n* = 2), progressive illness (*n* = 1), and administration errors (*n* = 6), were reported in 4% of participants, and 2% died during the course of treatment in the 11 studies with reported attrition rates. In selective reporting, missing domains in 14 studies (85% of participants) may have significantly biased some outcomes. Other biases in estimate of causal pathways assumed a direct causal link between serum levels and treatment successes based on assay samples alone. Three studies used active control agents (soybean or fluoride) and possibly biased outcomes (Papas et al., 2003; Lin et al., 2010; Lin et al., 2006). One study transferred four participants to the treatment group, potentially overcounting outcomes in the exposed group (misclassification differential bias) (Lambrecht et al., 2013). One study transferred patient files to the sponsor who then monitored outcomes (conflict of interest) (Buntzelet al., 2010). Figures 2 and 3 summarizes these findings in *Revman version 5.2* (Review Manager, 2014).

### Summary of trial and patient characteristics

The 16 included studies recruited 1120 participants, female to male ratio 1:2 in adults aged 18 to 83 years, mean age 49 years, and one study with children four to 18 years (Raphael et al., 2014). 40% included populations from Asiatic regions (Madan et al., 2008; Lin et al., 2010; Lin et al., 2006; Sangthawan, Phungrassami & Sinkitjarurnchai, 2013; Watanabe et al., 2010), 31% Europe (Markiewicz et al., 2012; Lambrecht et al., 2013; Buntzelet al., 2010; Raphael et al., 2014; Vokurka et al., 2005; Ertekin et al., 2004), 21% the Middle-East (Jahangard-Rafsanjani et al., 2013; Mansouri et al., 2012; Arbabi-kalati et al., 2012; Mehdipour et al., 2011), and 8% Anglo-America (Papas et al., 2003). Baseline characteristics were matched in nine studies with malignant neoplasms (head and neck cancer; all stages), and seven studies for all types of cancers and leukemia (Papas et al., 2003; Markiewicz et al., 2012; Raphael et al., 2014; Jahangard-Rafsanjani et al., 2013; Arbabi-kalati et al., 2012; Mehdipour et al., 2011; Vokurka et al., 2005). Tumor localizations and neutrophil count were unremarkable across studies. Nine percent of individuals exclusively underwent chemotherapy (Mansouri et al., 2012; Mehdipour et al., 2011), 22% radiotherapy (Madan et al., 2008; Sangthawan, Phungrassami & Sinkitjarurnchai, 2013; Ertekin et al., 2004), 29% chemo-radiotherapy (Lin et al., 2010; Lin et al., 2006; Lambrecht et al., 2013; Buntzelet al., 2010; Watanabe et al., 2010), 12% hematopoietic stem cell transplantation (HSCT) with or without specifying ALL or AML (refer to Table 2) (Papas et al., 2003; Arbabi-kalati et al., 2012), and 28% HSCT plus high dose chemotherapy conditioning (HDC) regimens (Markiewicz et al., 2012; Raphael et al., 2014; Jahangard-Rafsanjani et al., 2013; Vokurka et al., 2005). Chemotherapy combinations of unspecified dosages were reported in two studies as cisplatin, carboplatin, 5-fluorouracil, monoclonal antibody against epidermal growth factor receptor (EGFR), doxorubicin, dacarbazine, gemcitabine, methotrexate (Lambrecht et al., 2013; Arbabi-kalati et al., 2012). Radiation maximum exposures averaged between 60 to 70 Gy in all eight studies among individuals undergoing radiotherapy. HDC conditioning busulfan (4 mg/kg/d for 4 days; total 16 mg) and cyclophosphamide (60 mg/kg/d for 2 days; total 120 mg) were specified in two studies (Markiewicz et al., 2012; Jahangard-Rafsanjani et al., 2013) and treosulfan (42 g/m^2^) plus fludarabine (150 mg/m^2^) were specified in one study (Markiewicz et al., 2012).

**Table 2:** Trial and patient characteristics (see Appendix D, Table 1 for more details).

Study	*N*	Duration	Country	Mean age(range; years)	Sex(% female)	Baseline characteristics	Intervention	Comparator
Lambrecht et al., 2013	58	–	Belgium	56 (30–78)	72	Malignant neoplasms of the head and neck (all stages) and before undergoing chemotherapy and radiotherapy (first cycle); intervention at treatment start and before the onset of oral mucositis (OM).	Caphosol^®^ (Calcium phosphate) one minute rinse 15 ml, four to ten times daily plus standard care (Magic mouth wash, analgesics, antimycotics, antibiotics, parenteral tube) for 14 weeks (unspecified administration).	Standard care (Magic mouth wash, analgesics, fluconazole, antibiotics, parenteral tube) for 14 weeks (unspecific administration).
Raphael et al., 2014	34	–	Netherlands	11 (4–18)	44	Haematological malignancies and before undergoing Hematopoietic stem cell transplantation (HSCT) and chemotherapy (first cycle) at the onset of OM.	Caphosol^®^ (Calcium phosphate; unspecified usage) plus standard care (unspecified) self-administered for the duration of OM.	NaCl 0.9% mouth rinse (unspecified usage) plus standard care (unspecified) self-administered for the duration of OM.
Sangthawan, Phungrassami & Sinkitjarurnchai, 2013	139	3 months	Thailand	18 above	–	Histologically documented diagnosis of head and neck cancer (all stages); Kanofsky performance status at least 70 and before undergoing radiation therapy (1st cycle) before the onset of OM.	Zinc sulfate oral syrup (5 mg per 1cc) 10cc three times daily for five to seven weeks self-administered.	Placebo (unspecified components) three times daily for five to seven weeks self-administered.
Jahangard-Rafsanjani et al., 2013	77	21 days	Iran	32 (18–55)	43	Acute myeloid leukemia (AML) or acute lymphocytic leukemia (ALL) undergoing allogenic HSCT (cycle unspecified); Karnofsky performance status <70%; Intervention at HSCT start and before the onset of OM.	Selenium tablet (200 mcg) twice daily during transplantation and 14 days after (staff administered) plus standard care (20 drops of nystatin every three hours, chewable sucralfate tablet 500 mg every eight hours and mouth washes containing chlorhexidine 0.02% plus 10cc diluted povidone iodine every three hours).	Placebo tablet (unspecified) twice daily during transplantation and 14 days after (staff administered) plus standard care.
Markiewicz et al., 2012	40	–	Poland	37 (19–57)	40	AML or ALL or chronic myelogenous leukemia undergoing HSCT; intervention on the first day of conditioning before the onset of OM.	Calcium phosphate solution (equal volume, unspecified amount) four times daily self-administered until absolute neutrophil count ≥0.2 g/l.	Topical mouth care extract of salvia leaves twice daily, povidone-iodine mouth solution once daily, fluconazole mouth solution, glycerine (50 mg), vitamin A (10 g) and vitamin E (10 g) with or without benzocoaine (2.5 g) twice daily self-administered until absolute neutrophil count ≥ 0.2 g/l.
Mansouri et al., 2012	60	3 weeks	Iran	15 above	33	Hematologic malignancies undergoing high-dose chemotherapy conditioning regimen for allogenic HSCT (AML, ALL, CML, MDS) (cycle unspecified) intervention one day before conditioning and before the onset of OM.	Zinc sulfate capsule 220 mg (50 mg zinc elemental) twice daily 12-h intervals for three weeks administered by hospital staff.	Placebo capsules (unspecified components) twice daily in 1-hour intervals for three weeks administered by hospital staff.
Arbabi-kalati et al., 2012	50	20 weeks	Iran	49 (18–79)	48	Patients undergoing chemotherapy with same OM probability and Karnofsky performance ≥60 (1st cycle) intervention at treatment start and before the onset of OM.	Zinc sulfate capsule 220 mg three times daily self-administered until the end of chemotherapy.	Placebo capsule (similar shape, taste, color to intervention) three times daily self-administered until the end of chemotherapy.
Mehdipour et al., 2011	45	8 weeks	Iran	15 above	–	Hematological malignancies and acute myeloid leukemia undergoing chemotherapy (cycle unspecified); intervention start unspecified; before the onset of OM.	Zinc sulfate mouthwash (0.2% dilution) rinse twice daily for 14 days administered by an investigator.	–
Buntzelet al., 2010	39	7 years	Greece	63 (38.7–83)	20	Head and neck (squamous cell carcinoma) cancer undergoing chemoradiotherapy (cycle unspecified) intervention at two days before starting radiotherapy.	Sodium selenite oral fluid 500 µg one hour before radiotherapy; 300 µg during weekends and official holidays for unspecified length; unspecified administration.	No intervention.
Lin et al., 2010	97	12 months	Taiwan	52 (36–65)	20	Head and neck (nasopharangeal carcinoma) cancer or oral cancer (all stages) undergoing radiotherapy (recurrent or not); intervention start unspecified.	Oral zinc capsules (25 mg Pro-Z) three times daily for two months; unspecified administration.	Soybean oil capsules three times daily for two months; unspecified administration.
Watanabe et al., 2010	31	10 months	Japan	65 (35–78)	23	HNC (stage II–IV) undergoing radiotherapy or radiochemotherapy (cycle unspecified) intervention at treatment start.	Polaprezinc (Promac granules^®^ 15%) 0.5 g dissolved in 20 ml of 5% sodium alginate solution four times daily (three minute oral rinse then swallow) until end of radiotherapy;	Azulene (Azunol^®^ Gargle liquid 4%) in 100 ml water four times daily (three minute oral rinse no swallow) until end of radiotherapy; unspecified administration.
Madan et al., 2008	80	6 weeks	India	54 (18 above)	17	Head and neck malignancies (stage II–IV) undergoing radiotherapy (first cycle) intervention at treatment start before the onset of OM.	1% Povidone-iodine 10 ml mouthwash, twice daily for six weeks self-administered.	Plain water 10 ml mouthwash, twice daily for six weeks self-administered.
Lin et al., 2006	100	12 months	Taiwan	51 (39–62)	14	Head and neck cancer (stage I–IV) undergoing chemoradiotherapy (cycle unspecified) from first to last day of radiotherapy; OM status unspecified.	Zinc capsules (25 mg Pro-Z) three times daily for two months; unspecified administration.	Soybean oil capsules three times daily for two months; unspecified administration.
Vokurka et al., 2005	148	30 months	Czech Republic	54 (20–70)	39	Patients undergoing high dose chemotherapy and autologous peripheral stem cell transplantation (unspecified cycle); intervention start unspecified.	Povidone-iodine solution diluted 1:100 (Betadine 1 ml and 100 ml water for injection) mouth wash four times two minute gargle daily administered by study nurse for unspecified completion length.	Saline (NaCl 9% water solution) mouth wash four times, two minute gargle daily administered by study nurse for unspecified completion length.
Ertekin et al., 2004	27	13 weeks	Turkey	56 (18–71)	22	Head and neck cancer undergoing radiotherapy; (unspecified cycle); Karnofsky’s performance status ≥70; intervention at treatment start.	Zinc sulfate (50 mg Zinc; Zinco 220 capsule) three times daily at eight hour intervals during radiotherapy and six weeks after treatment; unspecified administration.	Empty placebo capsules taken three times daily at eight hour intervals during radiotherapy and six weeks after treatment; unspecified administration.
Papas et al., 2003	95	–	United States	43 (18–70)	6	HSCT (AML or ALL or chronic myelogenous leukemia, Hodgkin’s disease, non-Hodgkin’s lymphoma, multiple myelomas, myelodysplastic syndrome, breast cancer, ovarian cancer, other) (cycle unspecified); intervention start one week prior to treatment at screening.	Caphosol^®^ (Calcium phosphate) rinse four times daily, ten times daily when OM developed until engraftment and the resolution of OM; prior to HSCT four topical fluoride treatments of 1%F as neutral 2% NaF gel at screening; administered by trained unit nurses.	Aqueous sodium fluoride 0.01% 30 ml rinse four times daily, ten times daily when OM developed until engraftment and the resolution of OM; prior to HSCT four topical treatments with placebo gel administered by trained unit nurses.

Eleven studies (*n* = 841; 75%) administered the intervention before the onset of oral mucositis and when commencing cancer therapy (36% first cycle) (Madan et al., 2008; Lambrecht et al., 2013; Raphael et al., 2014; Sangthawan, Phungrassami & Sinkitjarurnchai, 2013; Arbabi-kalati et al., 2012) and all studies had the intention to treat (adjuvant or prophylactic) oral complications. Two studies were part of an ongoing study and imposed varying degrees of heterogeneity when compared to other studies (Lin et al., 2010; Vokurka et al., 2005). One study for children began the intervention at the onset of OM (Raphael et al., 2014). The intervention calcium phosphate was administered as the oral rinse Caphosol^®^ (dibasic sodium phosphate 0.032%, monobasic sodium phosphate 0.009%, calcium chloride 0.052%, sodium chloride 0.569%, purified water 30 ml) in three studies (Papas et al., 2003; Lambrecht et al., 2013; Raphael et al., 2014) four to ten times daily. One study used a supersaturated calcium phosphate rinse (SCPR) mixed in equal volumes calcium and phosphate aqueous solutions (Markiewicz et al., 2012). Zinc derivatives were zinc sulfate oral syrup 5 mg per cubic centimeter, three times daily (Sangthawan, Phungrassami & Sinkitjarurnchai, 2013); zinc sulfate capsules 50 mg zinc, two to three times daily (Mansouri et al., 2012; Arbabi-kalati et al., 2012; Ertekin et al., 2004); zinc sulfate mouthwash 0.2% oral rinse, twice daily (Mehdipour et al., 2011); zinc capsules 25 mg, three times daily (Lin et al., 2010; Lin et al., 2006); Polaprezinc (Promac granules^®^ 15%), four times daily (Watanabe et al., 2010). Selenium derivatives were administered as followed: selenium tablets 0.2 mg, twice daily (Jahangard-Rafsanjani et al., 2013); sodium selenite oral fluid 0.3 to 0.5 g an hour before radiotherapy sessions (Buntzelet al., 2010). Iodine was administered in the form Povidone-iodine 1% (betadine 1 ml) oral rinse two to four times daily (Madan et al., 2008; Vokurka et al., 2005), and one study used povidone-iodine as part of the standard care (see Table 2) (Markiewicz et al., 2012). Overall, 63% of the intervention was administered as oral rinse with the option of swallowing, and 37% in tablet form. Mineral derivative types were zinc derivatives (*n* = 549), calcium phosphate (*n* = 227), povidone-iodine (*n* = 228), and selenium (*n* = 116). There were three primary control conditions: standard care alone (*n* = 308), placebo capsules (*n* = 411), and placebo solutions (*n* = 401). OM severity was graded using variable scales: NCT-CTCAE (rated 0–5) assessed OM as adverse events in four studies (*n* = 262) (Lambrecht et al., 2013; Raphael et al., 2014; Sangthawan, Phungrassami & Sinkitjarurnchai, 2013; Watanabe et al., 2010); World Health Organization toxicity scale rated 0–4 in six studies (*n* = 455) (Markiewicz et al., 2012; Madan et al., 2008; Jahangard-Rafsanjani et al., 2013; Mansouri et al., 2012; Arbabi-kalati et al., 2012; Vokurka et al., 2005); four studies used a radiation toxicity scale- RTOG or ARMSC rated 0–4 (*n* = 263) (Lin et al., 2010; Lin et al., 2006; Buntzelet al., 2010; Ertekin et al., 2004); one study measured OM lesions using a Spijkevet scale rated 0–4 (*n* = 45) (Mehdipour et al., 2011); and a dental scale-NIDCR rated 0–5 (*n* = 95) (Papas et al., 2003). Visual Analog Scales were used to measure pain ranging from 0–5, 0–10, or 0–100. All outcomes were measured in days.

### Oral mucositis severity outcomes

**Figure 4:**
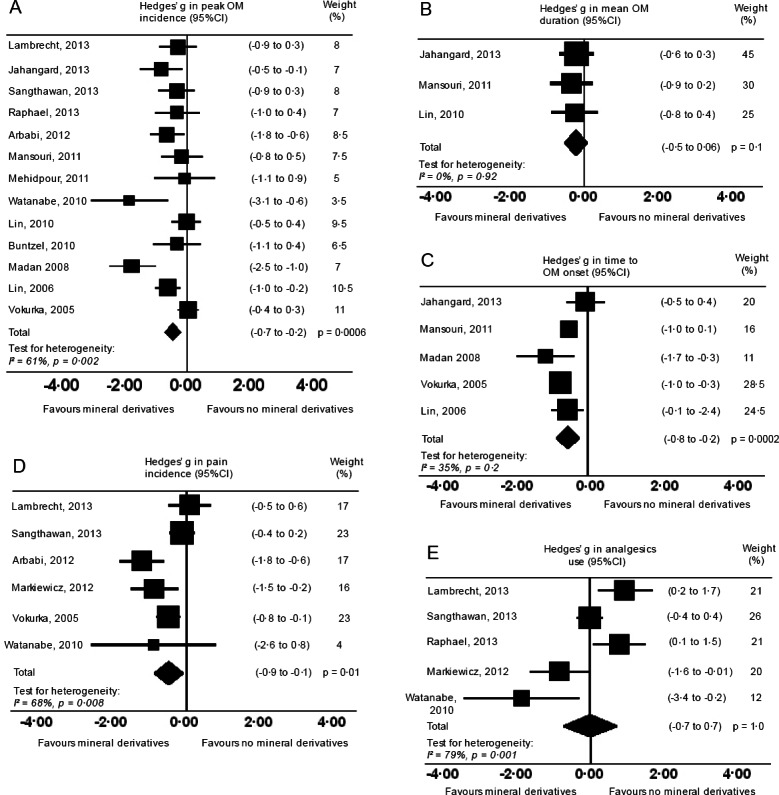
Forest plots. Forest plots on severity (primary) outcomes: (A) Peak OM incidence. (B) OM mean duration. (C) Time to OM onset. Mineral derivative treatment groups were less likely to experience peak OM and time to OM onset delayed. OM mean duration in days was not significantly different. Forest plots on secondary outcomes: (D) Incidence of pain. (E) Analgesics use. Incidences of pain and analgesics use were not significant.

Of sixteen studies, thirteen studies reported OM peak incidence across OM severity grading systems (Fig. 4A). Two studies did not experience peak OM and were therefore excluded (Markiewicz et al., 2012; Ertekin et al., 2004). Another study imposed significant heterogeneity when included in the analysis (from *I*^2^ = 61% to *I*^2^ = 87%), possibly from a confounder (fluoride as part of standard care), and was therefore excluded (Papas et al., 2003). Participants who undertook mineral derivatives were less likely to experience peak OM than those without treatment (*g* = −0.47, 95% CI −0.7 to −0.2, *p* = 0.0006, *I*^2^ = 61%). Despite high heterogeneity, spurious conditions were expected in underpowered studies, hence all studies were kept in the analysis for observing a general effect trend. OM mean duration were reported in six studies (Fig. 4B). However, three were excluded since substitute data artefacts (see Appendix C) caused marked heterogeneity (from *I*^2^ = 0% to *I*^2^ = 97%, *I*^2^ = 82%, *I*^2^ = 45%) (Papas et al., 2003; Markiewicz et al., 2012; Raphael et al., 2014). OM mean durations did not significantly differ between mineral derivative and control groups (*g* = −0.2, 95% CI 0.1 to −0.5, *p* = 0.128, *I*^2^ = 0%). Conversely, times to OM onset reported in five studies were significantly delayed in treated participants (*g* = −0.5, 95% CI −0.8 to −0.2, *p* = 0.0002, *I*^2^ = 35%; Fig. 4C) (Madan et al., 2008; Lin et al., 2006; Jahangard-Rafsanjani et al., 2013; Mansouri et al., 2012; Vokurka et al., 2005).

### Pain and analgesics use

Six studies reported incidences of pain using a visual analog scale (0–5 or 0–10) (Markiewicz et al., 2012; Lambrecht et al., 2013; Sangthawan, Phungrassami & Sinkitjarurnchai, 2013; Arbabi-kalati et al., 2012; Watanabe et al., 2010; Vokurka et al., 2005). One study caused marked heterogeneity (from *I*^2^ = 68% to *I*^2^ = 96%) possibly from a different visual analog scale (0–100), and a confounder (fluoride as part of standard care) and hence was excluded in the analysis (Papas et al., 2003). Experiencing pain was less likely in treated participants (*g* = −0.5, 95% CI −0.1 to −0.9, *p* = 0.01, *I*^2^ = 68%; Fig. 4D). However, pains experienced during cancer therapies were unlikely estimates of OM pain in isolation—an explanation as to why analgesic use was no different across groups (*g* = −0.01, 95% CI 0.7 to −0.7, *p* = 0.977, *I*^2^ = 79%), and significant heterogeneity apparent in both pain incidences and analgesic use (Fig. 4E). One study alone did not significantly alter the sensitivity in analgesics use or in the six pain studies. Thus it was assumed included studies did not record pain or analgesics use at uniform time points, or measured OM pain in isolation from other cancer therapy pains.

### Serum mineral levels (inflammation and infection)

Serum samples were significantly noted in seven studies (Papas et al., 2003; Lin et al., 2010; Lin et al., 2006; Buntzelet al., 2010; Sangthawan, Phungrassami & Sinkitjarurnchai, 2013; Jahangard-Rafsanjani et al., 2013; Vokurka et al., 2005). Serum selenium levels were measured in all selenium studies, and cancer therapy toxicities were less in treatment than control groups (stomatitis-less toxicity treatment 36.4% versus 23.5% control (Wilson, 2014); treatment 118.21 versus 94.78 nmol/min/mL plasma Glu.Px-scavenger radical for alleviating toxicities) (Buntzelet al., 2010; Mansouri et al., 2012). In three studies, zinc serum levels were also higher in zinc treatment groups than control groups (63 to 142 ± 31 µg/dl versus 69 to 117 ± 25 µg/dl post treatment serum zinc level) (Lin et al., 2010; Lin et al., 2006; Sangthawan, Phungrassami & Sinkitjarurnchai, 2013). Pro-inflammatory cytokines TNF*α* and Interleukins (*IL-1, IL-2* and *IL-6*) reportedly play a role in developing OM, and formed a basis for measuring serum levels in two studies (selenium; zinc) (Jahangard-Rafsanjani et al., 2013; Watanabe et al., 2010). One of those studies formulated the treatment ‘polaprezinc’ based on reports it inhibited cellular signaling of TNF*α* but the study did not record serum levels (Watanabe et al., 2010). Infection and oral bacteria flora were observed in two zinc studies by the same author (Lin et al., 2010; Lin et al., 2006), and were also mentioned in one calcium phosphate study using microbiology tests for detecting *S. mutans and Lactobacillus. S. mutans* (Papas et al., 2003). There were also three excluded studies (Appendix D, Table 2) that measured bacteria in saliva samples but were not included in the review because the causal pathway on developing OM and oral bacteria was unclear, and posed a high risk of bias (Ertekin et al., 2003; Nagy et al., 2000; Meca et al., 2009).

### Total Parental Nutrition (TPN) use

TPN were recorded in two calcium phosphate studies. In one study, there were no significant differences in the treatment and control groups (parental feeding *p* = 0.7; tube feeding *p* = 0.08; age range 4–18 years) (Raphael et al., 2014). In the other study, less TPN were used by treatment than control groups in an adult population (*p* = 0.02; 19–57 years) (Markiewicz et al., 2012).

### Adverse events

Three studies discussed dysphagia (difficulty swallowing) in conjunction with oral mucositis scoring systems (Markiewicz et al., 2012; Lambrecht et al., 2013; Buntzelet al., 2010). In two calcium phosphate studies, there were no significant differences in severity between treatment and control groups (Markiewicz et al., 2012; Lambrecht et al., 2013). However, the selenium group had a significantly lower mean severity score of 1.5 versus 2.2 in the control group (RTOG radiation scale 0–4) (Buntzelet al., 2010). The same study did not observe a significant difference in xerostomia (dry mouth) and ageusia (taste loss). In one zinc study, xerostomia (*p* = 0.007; grade ≥2 CTCAE) and ageusia (*p* = 0.006; grade ≥2 CTCAE adverse events scale 0–5) showed significant improvement in treatment than control groups (Watanabe et al., 2010). Xerostomia also improved in the zinc treatment groups (*p* = 0.005; WHO criteria 0–4 adverse events) (Arbabi-kalati et al., 2012). Nausea and vomiting recorded in one zinc study were mostly mild, and abdominal pain, diarrhea, fever, lethargy did not occur in treatment groups (Sangthawan, Phungrassami & Sinkitjarurnchai, 2013). Neutrophil count and neutropenia were reported in three calcium phosphate studies but were not significant (Papas et al., 2003; Markiewicz et al., 2012; Raphael et al., 2014).

### Publication bias

**Figure 5:**
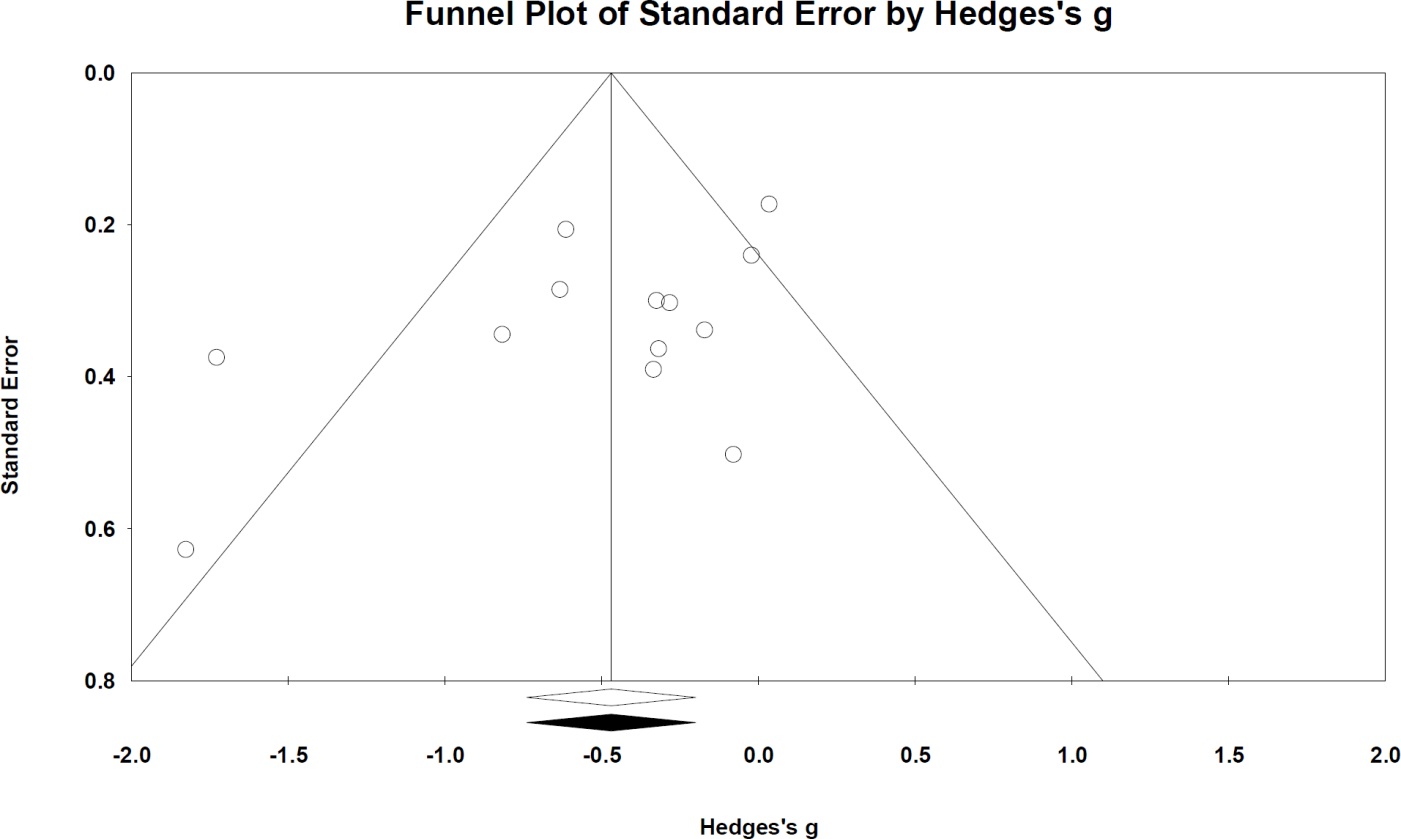
Funnel plot based on peak OM incidence across study publications (*n* = 13 studies). Egger’s test *p* = 0.07. Publication bias was not significant.

Publication bias was assessed for all outcomes but evidence of bias was not significant. Funnel plots by and large appeared symmetric—assuming outliers (outside funnel) were small study effects from small sample sizes. Egger’s test was also not significant: Peak OM incidence *p* = 0.07 (Fig. 5); OM mean duration *p* = 0.5; time to OM onset *p* = 0.9; pain incidence *p* = 0.4; analgesics use *p* = 0.7.

### Decision tree

**Figure 6:**
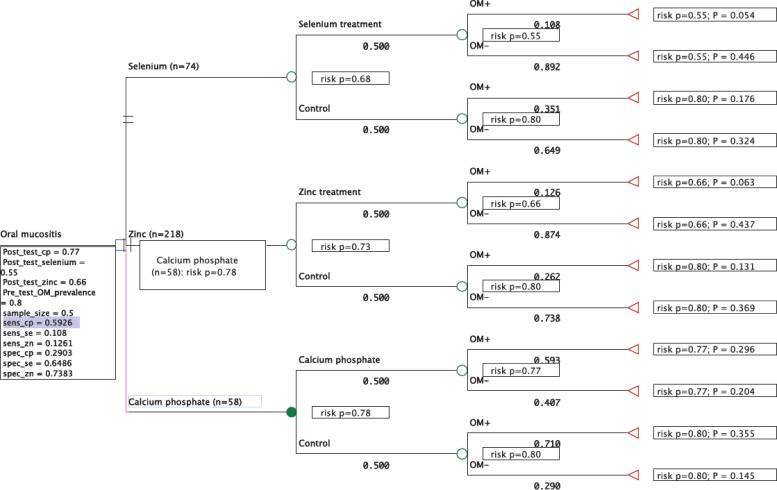
A decision tree “rolled back” in arriving at the most viable (risk-averse and effective) option. “*P*” denotes probability values for each treatment arm totaling one (three in total).

One study was included for calcium phosphate based on available raw data and a low risk of bias (*n* = 58) (Lambrecht et al., 2013); another study for selenium (*n* = 74) (Jahangard-Rafsanjani et al., 2013) under the same assumptions; three studies for zinc (*n* = 218) (Sangthawan, Phungrassami & Sinkitjarurnchai, 2013; Mansouri et al., 2012; Watanabe et al., 2010); povidone-iodine was not included due to high bias. The decision analysis suggests the calcium phosphate treatment groups were the most at risk of developing OM at 78% (close to 80% pre-treatment rate) followed by zinc 73%, and selenium 68%. The probable outcome between each arm (rate of true positives, false positives, true negatives and false negatives) at present does not reflect a recognizable trend (Fig. 6).

## Discussion

The results suggest mineral-derivatives have a positive effect in alleviating the severity of oral mucositis during cancer therapy. Its effect on pain is unclear. This review combines mineral derivatives in observing a general effect trend, and supports earlier reviews demonstrating the positive effects of specific mineral derivatives (chiefly zinc sulfate) in prevention and treatment of oral mucositis (Worthington et al., 2013). Surprisingly, there were no recent trials on the effects of magnesium sulfate on oral mucositis during recent cancer therapies, even though its effect in alleviating toxicities and adverse events are often reported (Kumar, 2012). This review is limited in recommending which definitive mineral derivative alleviates oral mucositis exclusively. Other limitations include high heterogeneity implicated by variable conditionals and prior’s i.e., different protocols and diverse cancer therapies. Also a ‘placebo effect’ may have undermined non-blinded studies at high risk of bias. For instance, participants may have reported positive outcomes when socially primed on study aims (Lutfey, 2013). Other limitations include an unclear causal link between oral mucositis and serum levels for detecting inflammation and infection via cell signals. The prospects of developing oral mucositis anti-viral agents or immunosuppressants are considered novel. Emerging clinical trials appear to target the immune system with *IL-7* vaccines (National Cancer Institute, 2000a). At present, quantifying and translating serum levels and cell signal parameters to OM grading systems, and include these figures in the meta-analysis triggers significant bias.

Adverse events were largely unremarkable in reported studies. Adverse event scales were different to toxicity scales but there was insufficient data to explore this further. Data constraints were also seen in the use of TPN in children or adults. It was unclear if TPN was used for rescue therapy or part of the standard care. Evidence of publication bias was not detected, but if baseline characteristics were matched across studies with a complete dataset, publication bias in high risk of bias groups may have been detected. However, there was an equal spread of mineral derivatives effective or null across all studies, hence publication bias in this review seems unlikely.

The findings in this review do not appear robust for making clear recommendations, nor are they translatable for clinical practice. The predominantly small sample sizes conducted in single-centers make it difficult to make these findings generalizable. This review at present and in practice may serve a likely indicator in considering electrolyte treatments of known mineral-derivative efficacy. In this context, future studies may include zinc, calcium, iodine, or selenium as electrolytes gargled or ingested alongside magnesium sulfate during cancer therapies. Trials on electrolyte balance in cancer therapies remain scant (Chernecky, Murphy-Ende & Macklin, 2006). Current trials on magnesium sulfate combinations i.e., with calcium gluconate, target cancer therapy-induced toxicities (National Cancer Institute, 2000b). However, this link between toxicities and adverse events remains unclear and produced a high risk of bias in this review. Therefore, there is added uncertainty on whether combination minerals administered intravenously reduces the incidence of oral mucositis exclusively (Rosner & Dalkin, 2014). Future trials should consider serum levels and computer simulations when designing mineral tolerance thresholds to weigh the benefits and harms (Selvin, 2011). This may include decision trees with probable risks. When meta-analyses map to decision trees, it compares treatment arms and generates probabilities that could further assess the benefits and harms. These are future avenues that could test the efficacy of mineral derivatives in treating oral mucositis using safe, practicable, and cost-effective strategies.

## Supplemental Information

10.7717/peerj.765/supp-1 Appendix A Search strategies Appendix A: Search strategies

10.7717/peerj.765/supp-2 Appendix B Template

10.7717/peerj.765/supp-3 Appendix C Dealing with missing data Appendix C: Dealing with missing data

10.7717/peerj.765/supp-4 Appendix D Characteristics of studies Appendix D

10.7717/peerj.765/supp-5 Supplemental Information PRISMA checklist

## Additional Information and Declarations

### Data Deposition

The following information was supplied regarding the deposition of related data: Link to data and appendices: http://openwetware.org/wiki/User:Sonia_Lee/Notebook/minerals4cancer.
