# Supplementary material for: Mineral derivatives in alleviating oral mucositis during cancer therapy: a systematic review
Source: PeerJ. 2015 Feb 12;3:e765. doi: 10.7717/peerj.765 (PMC4330907; doi:10.7717/peerj.765)
Supplement: Appendix A — Search strategies [file peerj-03-765-s001.doc]

Appendix A: Search strategies

*MEDLINE via OVID search strategy*

1. (Exp minerals or mineral$) or (exp antioxidants or antiox$) or ((exp Diet or diet$) AND (exp minerals or mineral$)) or (exp micronutrients or micronutrient$) or (exp trace elements or trace element$) or (exp dietary supplements or diet$ AND supplement$) or macromineral$ or micromineral$ or (exp calcium or exp calcium sulphate or calcium$ or gypsum$) or (exp selenium or selen$) or (exp magnesium or magnesi$) or (exp zinc or zinc$) or (exp iron or exp ferrous compounds or iron$ or ferrous$) or ((exp iodine or exp iodine compounds or iodi$) NOT (exp iodine isotopes or exp iodine radioisotopes)) or ((exp copper or exp copper sulfate or copper$ or cupric$ or pentahydrate$ or trihydrate$) NOT (exp copper radioisotopes)) or (exp manganese or exp manganese compounds or manganese$) or (exp fluorides or fluoride$) or ((exp chromium or exp chromium compounds or chromium$) NOT (exp chromium isotopes or exp chromium radioisotopes or exp chromium alloys)) or (exp molybdenum or molybden$)
2. exp stomatitis or exp candidiasis, oral or exp mucositis or(stomatitis or mucositis or (oral and candi$) or (oral and fung$) or mycosis or mycotic).mp.
3. (neoplasm$ or cancer$ or chemo$).mp.
4. (randomized controlled trial or controlled clinical trial).pt. or (randomized or placebo or randomly or trial or groups).ab. or drug therapy.fs.
5. (animals/ not humans/) or (review or meta-analysis).pt.
6. (1 and 2 and 3 and 4) not 5
7. limit 6 to yr="2000 -Current"

*Embase search strategy*

1. ‘mineral’/exp OR mineral* AND [2000-2014]/py
2. ‘antioxidant’/exp OR antiox* AND [2000-2014]/py
3. (‘diet’/exp OR diet*) AND (‘mineral’/exp OR mineral*) AND [2000-2014]/py
4. ‘micronutrients’/exp OR micronutrient* AND [2000-2014]/py
5. ‘trace elements’/exp OR trace element* AND [2000-2014]/py
6. ‘dietary supplements’/exp OR (diet* AND supplement*) AND [2000-2014]/py
7. macromineral* OR micromineral* AND [2000-2014]/py
8. ‘calcium’/exp OR ‘calcium sulfate’/exp OR calcium* or gypsum* AND [2000-2014]/py
9. ‘selenium’/exp or selen* AND [2000-2014]/py
10. ‘magnesium’/exp OR magnesi* AND [2000-2014]/py
11. ‘zinc’/exp OR zinc* AND [2000-2014]/py
12. ‘iron’/exp OR ‘ferrous compounds’/exp OR iron* OR ferrous* AND [2000-2014]/py
13. (‘iodine’/exp OR ‘iodine compounds’/exp or iodi*) NOT (‘iodine isotopes’/exp OR ‘iodine radioisotopes’/exp) AND [2000-2014]/py
14. (‘copper’/exp OR ‘copper sulfate’/exp OR copper* OR cupric* OR pentahydrate* OR trihydrate*) NOT (‘copper radioisotopes’/exp) AND [2000-2014]/py
15. ‘manganese’/exp OR ‘manganese compounds’/exp OR manganese* AND [2000-2014]/py
16. ‘fluorides’/exp or fluoride* AND [2000-2014]/py
17. (‘chromium’/exp OR ‘chromium compounds’/exp OR chromium*) NOT (‘chromium isotopes’/exp OR ‘chromium radioisotopes’/exp OR ‘chromium alloys’/exp) AND [2000-2014]/py
18. ‘molybdenum’/exp OR molybden* AND [2000-2014]/py
19. or/#1 to #18
20. ‘stomatitis’/exp AND [2000-2014]/py
21. thrush* AND [2000-2014]/py
22. ‘mucositis’/exp AND [2000-2014]/py
23. oral* AND candidiasis* AND [2000-2014]/py
24. oral* AND fung* AND [2000-2014]/py
25. ‘mycosis’/exp OR ‘mycotic’ AND [2000-2014]/py
26. or/#20 to #25
27. ‘neoplasm’/exp OR neoplasm* AND [2000-2014]/py
28. ‘cancer’/exp OR cancer* AND [2000-2014]/py
29. ‘chemotherapy’/exp OR chemo* AND [2000-2014]/py
30. or/#27 to #29
31. crossover*:ab,ti AND [2000-2014]/py
32. cross AND over*:ab,ti AND [2000-2014]/py
33. ‘cross over’:ab,ti AND [2000-2014]/py
34. placebo*:ab,ti AND [2000-2014]/py
35. ‘placebo’/exp AND [2000-2014]/py
36. doubl* AND blind*:ab,ti AND [2000-2014]/py
37. allocat*:ab,ti AND [2000-2014]/py
38. random* AND [2000-2014]/py
39. or/#31-38
40. ‘animal’/exp OR ‘nonhuman’/exp OR animal AND ‘experiment’/exp
41. ‘human’/exp AND [2000-2014]/py
42. #40 AND #41 AND [2000-2014]/py
43. #40 NOT #42 AND [2000-2014]/py
44. #39 NOT #43 AND [2000-2014]/py
45. ‘review’/exp OR ‘meta-analysis’/exp AND [2000-2014]/py
46. #19 AND #26 AND #30 AND #44 NOT #45 AND [embase]/lim AND [2000-2014]/py

*CENTRAL search strategy*

1. (Exp minerals or mineral$) or (exp antioxidants or antiox$) or ((exp Diet or diet$) AND (exp minerals or mineral$)) or (exp micronutrients or micronutrient$) or (exp trace elements or trace element$) or (exp dietary supplements or diet$ AND supplement$) or macromineral$ or micromineral$ or (exp calcium or exp calcium sulphate or calcium$ or gypsum$) or (exp selenium or selen$) or (exp magnesium or magnesi$) or (exp zinc or zinc$) or (exp iron or exp ferrous compounds or iron$ or ferrous$) or ((exp iodine or exp iodine compounds or iodi$) NOT (exp iodine isotopes or exp iodine radioisotopes)) or ((exp copper or exp copper sulfate or copper$ or cupric$ or pentahydrate$ or trihydrate$) NOT (exp copper radioisotopes)) or (exp manganese or exp manganese compounds or manganese$) or (exp fluorides or fluoride$) or ((exp chromium or exp chromium compounds or chromium$) NOT (exp chromium isotopes or exp chromium radioisotopes or exp chromium alloys)) or (exp molybdenum or molybden$)
2. MeSH descriptor neoplasms explode all trees
3. neoplasm* or cancer* or chemo*
4. MeSH descriptor stomatitis explode all trees
5. MeSH descriptor mucositis explode all trees
6. MeSH descriptor candidiasis explode all trees
7. stomatitis
8. (Stevens next Johnson syndrome)
9. mucositis
10. oral near cand*
11. mouth near cand*
12. oral and fung*
13. mouth and fung*
14. (mycosis or mycotic or thrush)
15. #4 or #5 or #6 or #7 or #8 or #9 or #10 or #11 or #12 or #13 or #14
16. #1 AND #3 AND #15
17. review
18. #16 not #17

*CANCERLIT (PubMed Cancer Subset) search strategy*

((((((((((((minerals [mh]) or (antioxidants [mh] or antiox*) or ((Diet [mh]) AND (minerals [mh])) or (micronutrients [mh] or micronutrient*) or (trace elements [mh] or trace element*) or (dietary supplements [mh] or diet* AND supplement*) or macromineral* or micromineral* or (calcium [mh] or calcium sulphate [mh] or calcium* or gypsum*) or (selenium [mh] or selenium*) or (magnesium [mh] or magnesi*) or (zinc [mh] or zinc*) or (iron [mh] or ferrous compounds [mh] or iron* or ferrous*) or ((iodine [mh] or iodine compounds [mh] or iodi*) NOT (iodine isotopes [mh] or iodine radioisotopes [mh])) or ((copper [mh] or copper sulfate [mh] or copper* or cupric* or pentahydrate* or trihydrate*) NOT (copper radioisotopes [mh])) or (manganese [mh] or manganese compounds [mh] or manganese*) or (fluorides [mh] or fluoride*) or ((chromium [mh] or chromium compounds [mh] or chromium*) NOT (chromium isotopes [mh] or chromium radioisotopes [mh] or chromium alloys [mh])) or (molybdenum [mh] or molybden*)))) AND ((neoplasm* or chemotherapy*))) AND ((stomatitis OR “Stevens Johnson syndrome” OR “candidiasis oral” OR mucositis OR (oral AND (candid* OR mucos* OR fung*)) OR mycosis OR mycotic OR thrush))) AND ((randomized controlled trial [pt] OR controlled clinical trial [pt] OR randomized controlled trials [mh] OR random allocation [mh] OR double-blind method [mh] OR single-blind method [mh] OR clinical trial [pt] OR clinical trials [mh] OR (“clinical trial” [tw] OR ((singl* [tw] OR doubl* [tw] OR trebl* [tw] OR tripl* [tw]))) AND ((mask* [tw] OR blind* [tw] )) OR (placebos [mh] OR placebo* [tw] OR random* [tw] OR research design [mh:noexp]))) NOT ((animals [mh] NOT human [mh]))) NOT (review [pt] OR meta-analysis)))))

Filters

1. Date range: 2000/01/01-2014/09/11

2. Humans

*CINAHL via EBSCO search strategy*

S1 (MH “Minerals+”) OR Mineral*
S2 (MH “Dietary Supplements+”) OR (MH “Therapeutics+”) OR (Diet* AND Suppl*)
S3 (MH “Antioxidants+”) OR Antiox*
S4 (MH “Micronutrients+”) OR Micronutrient*
S5 (MH “Trace Elements+”) OR (Trace element*)
S6: (MH “Transition Elements+”) OR (Transition element*)
S7: (MH “Free radicals+”)
S8 (MH “Calcium Compounds+”) OR Calcium* OR (Calcium sulfate*) OR Gypsum*
S9 (MH “Selenium Compounds+”) OR Selen*
S10 (MH “Magnesium Compounds+”) OR Magnesi*
S11 (MH “Zinc Compounds+”) OR Zinc*
S12 (MH “Iron Compounds+”) OR Iron* OR Ferrous*
S13 (MH “Iodine Compounds+”) OR Iodi*
S14 (MH “Copper+”) OR Copper* OR (Copper sulfate*) OR Cupric* OR Pentahydrate* OR Trihydrate*
S15 Manganese*
S16 (MH “Fluorine Compounds+”) OR Fluoride*
S17 Chromium*
S18 (MH “Molybdenum+”) OR Molybden*
S19 NOT (MH “Radioisotopes+”) OR Radioisotope*
S20 NOT (MH “Isotopes+”) OR Isotope*
S21 NOT (MH “Alloys+”) OR Alloy*
S22 Or/S1-S18 NOT (S19-21)
S23 (MH “Stomatitis+”) OR Stomatitis*
S24 (MH “Mucositis+”) OR Mucositis*
S25 (MH “Mouth Neoplasms+”)
S26 (MH “Candidiasis, Oral+”) OR (Oral* AND Candi*)
S27 (Oral* AND Fung*)
S28 Mycosis* OR Mycotic*
S29 Or/S23-28
S30 (MH “Neoplasms+”) OR Neoplasm*
S31 (MH “Chemotherapy, Cancer+”) OR Chemo*
S32 (MH “Chemotherapy, Adjuvant+”)
S33 (MH “Antineoplastic Agents+”)
S34 Or/30-35
S35 #S22 AND #S29 AND #S34

Filters

1. January 2000 – September 2014
2. Exclude Medline records
3. Human
4. Randomized Controlled Trial
