# Supplementary material for: Mineral derivatives in alleviating oral mucositis during cancer therapy: a systematic review
Source: PeerJ. 2015 Feb 12;3:e765. doi: 10.7717/peerj.765 (PMC4330907; doi:10.7717/peerj.765)
Supplement: Appendix B [file peerj-03-765-s002.doc]

Appendix B: Abstraction template

| **Mineral Derivatives for chemo-induced oral mucositis** |
| --- |

Reviewer + Date: Include ☐Exclude ☐

| **Report Title** |  | | | | | | | | | | | | | | | | | | |
| --- | --- | --- | --- | --- | --- | --- | --- | --- | --- | --- | --- | --- | --- | --- | --- | --- | --- | --- | --- |
| Trial name/code |  | | | | | | | | | | | | | | | | | | |
| First author |  | | | | | | | | Year of publication | | | | | | | | | | |
| Source/Journal |  | | | | | | | | | | | | | | | | | | |
| Publication type |  | | | | | | | | Database | | | | | | | | | | |
| Country |  | | | | | | | | Duration | | | | | | | | | | |
| Population | N =  Intervention: n = Control: n =  Other: | | | Age: | | | | | | | | | | | Female: | | | | |
| Rationale for intervention | Chemotherapy-adjuvant (maintenance) Prophylaxis (prevention)  Rescue therapy Other: | | | | | | | | | | | | | | | | | | |
| Diagnosis | Cancer type: | | | | | | | | Chemo-treatment: | | | | | | | | | | |
| Oral condition | Type: | | | | | | | | Onset: | | | | | | | | | | |
| Type of study | Randomised  Cross-over Parallel Other _______________  1:1 1:1:1 Other ratio: ___________________  Placebo condition: __________________________  Arms: ____________________________________ | | | | | | | | Quasi randomised | | | | | | | | | | |
| Intervention Drug (mg/ml) Dosage cycle | Zinc | Calcium | | | | | | | Fluoride | | | | | | | Other | | | |
| Comparator | 1. | | | | | 1. | | | | | | | | | | 1. | | | |
|  | 2. | | | | | 2. | | | | | | | | | | 2. | | | |
|  | 3. | | | | | 3. | | | | | | | | | | 3. | | | |
| Confounder/s (Primary) | 1. | | | | | | | | | | | | | | | | | | |
|  | 2. | | | | | | | | | | | | | | | | | | |
|  | 3. | | | | | | | | | | | | | | | | | | |
|  | 4. | | | | | | | | | | | | | | | | | | |
| Endpoints | Primary: 1 2 3 4 5 0 | | | | | | | | | | Secondary: 1 2 3 4 5 0 | | | | | | | | |
| Types of outcome measures |  | | Time of reporting | | | | | | | | | | | | | | | | |
|  |  |  | 1d | | 2d | | 3d | 4d | | 5d | | 6d | 7d | 21d | | | 1m | QoL | Other |
|  | No mouth sores | |  | |  | |  |  | |  | |  |  |  | | |  |  |  |
|  | Reduction in mouth sores | |  | |  | |  |  | |  | |  |  |  | | |  |  |  |
|  | Reoccurrence | |  | |  | |  |  | |  | |  |  |  | | |  |  |  |
|  | Complications | |  | |  | |  |  | |  | |  |  |  | | |  |  |  |
|  | Treatment adverse reaction | |  | |  | |  |  | |  | |  |  |  | | |  |  |  |
|  | QALYs | |  | |  | |  |  | |  | |  |  |  | | |  |  |  |
|  |  |  |  |  |  |  |  |  |  |  |  |  |  |  |  |  |  |  |  |
|  |  |  |  |  |  |  |  |  |  |  |  |  |  |  |  |  |  |  |  |
|  |  |  |  |  |  |  |  |  |  |  |  |  |  |  |  |  |  |  |  |
|  |  |  |  |  |  |  |  |  |  |  |  |  |  |  |  |  |  |  |  |
|  |  |  |  |  |  |  |  |  |  |  |  |  |  |  |  |  |  |  |  |
|  |  |  |  |  |  |  |  |  |  |  |  |  |  |  |  |  |  |  |  |
|  |  |  |  |  |  |  |  |  |  |  |  |  |  |  |  |  |  |  |  |

**Comments:**
