# Supplementary material for: Mineral derivatives in alleviating oral mucositis during cancer therapy: a systematic review
Source: PeerJ. 2015 Feb 12;3:e765. doi: 10.7717/peerj.765 (PMC4330907; doi:10.7717/peerj.765)
Supplement: Appendix C — Dealing with missing data [file peerj-03-765-s003.doc]

Appendix C: Dealing with missing data

|  | Missing data | Resolution |
| --- | --- | --- |
| Lambrecht, 2013^27^ | - OM onset  - OM duration | Excluded outcome comparisons: time to OM onset and OM duration (no data). |
| Jahangard, 2013^31^ | - Analgesic use  - Pain incidence | Excluded outcome comparisons: use of analgesics and incidence of pain (no data). |
| Sangthawan, 2013^30^ | - OM onset  - OM duration | Excluded outcome comparisons: time to OM onset and OM duration (no data). |
| Raphael, 2013^29^ | - OM onset  - Pain incidence | Excluded outcome comparisons: time to OM onset and incidence of pain (no data). |
| Arbabi, 2012^33^ | - Peak OM  - OM onset  - Analgesics use | Peak OM incidence: calculated overall mean score and mean standard deviation from weekly scores: zinc group 1·468±0·3896(n=25); control group 2·106±0·2283(n=25).  Excluded outcome comparisons: time to OM onset and analgesic use (no data). |
| Markiewicz, 2012^23^ | - Peak OM  - OM onset  - OM duration  - Pain incidence | Peak OM incidence: treatment and control did not experience peak OM (no data). Excluded.  OM onset: Excluded (no data).  OM duration: missing standard deviations – imputed ±2 (treatment and control) estimate from studies with similar OM durations.  Pain incidence: missing standard deviations – imputed ±1 (treatment and control) estimate from studies with similar pain incidence. |
| Mansouri, 2011^32^ | - OM onset  - OM duration  - Analgesics use  - Pain incidence | OM onset: missing standard deviations – imputed ±1·5 (treatment and control) estimate from studies with similar OM onset.  OM duration: missing standard deviations – imputed ±2 (treatment and control) estimate from studies with similar OM duration.  Use of analgesics: Excluded (no data).  Pain incidence: Excluded (no data). |
| Mehdipour, 2011^34^ | - Peak OM  - OM onset  - OM duration  - Analgesics use  - Pain incidence | Peak OM incidence: converted ANOVA p-value=0·025 to d=0·0816, v=0.2669 (Practical meta-analysis effect size calculator).  Excluded outcome comparisons: time to OM onset, OM duration, use of analgesics and incidence of pain (no data). |
| Watanabe, 2010^35^ | - OM onset  - OM duration  - Pain incidence | Excluded outcome comparisons: OM onset, OM duration and incidence of pain (no data). |
| Lin, 2010a^25^ | - OM onset  - Analgesics use  - Pain incidence | Excluded outcome comparisons OM onset, analgesics use and incidence of pain (no data). |
| Buntzel, 2010^28^ | Excluded from meta-analysis. | Discussed. High bias and heterogeneity. |
| Madan, 2008^24^ | - Peak OM  - OM onset  - OM duration  - Analgesics use  - Pain incidence | Peak OM incidence: calculated overall mean score and mean standard deviation from weekly score:  Iodine group 1·157±0·5659(n=18); control group 2·233±0·7922 (n=20).  OM onset: converted chi-square p-value=28·32 to d=1·023, v=0·1328 (Practical meta-analysis effect size calculator).  Excluded outcome comparisons OM duration, analgesics use and incidence of pain. |
| Lin, 2006b^26^ | - Peak OM  - OM onset  - Analgesics use  - Pain incidence | Peak OM: converted log rank p-value-0·003 to d=0·6186, v=0·04321 (Practical meta-analysis effect size calculator).  OM onset: converted log rank p-value=0·017 to d=0·4934, v=0·042496 (Practical meta-analysis effect size calculator).  Excluded outcome comparisons analgesics use and incidence of pain. |
| Vokurka, 2005^36^ | - Peak OM  - OM onset  - OM duration  - Analgesics use  - Pain incidence | Peak OM: decided on grade III over grade IV cumulative scores, under the assumption participants with grade IV initially experienced grade III. Converted t-test p-value=0·84 to d=0·0352, v=0·030315 (Practical meta-analysis effect size calculator).  OM onset: missing standard deviations – imputed ±1·5 (treatment and control) estimate from studies with similar OM onset.  Excluded outcome comparisons OM duration and analgesics use (no data).  Incidence of pain: missing standard deviations – imputed ±1 (treatment and control) estimate from studies with similar incidence of pain. |
| Ertekin, 2004^37^ | Excluded from meta-analysis. | Discussed. None experienced peak OM. No data for outcome comparisons. |
| Papas, 2003^22^ | Excluded from meta-analysis. | Discussed. Significantly different scale for outcome comparisons. High heterogeneity. |
