# Supplementary material for: Mineral derivatives in alleviating oral mucositis during cancer therapy: a systematic review
Source: PeerJ. 2015 Feb 12;3:e765. doi: 10.7717/peerj.765 (PMC4330907; doi:10.7717/peerj.765)
Supplement: Appendix D [file peerj-03-765-s004.doc]

Appendix D

**Summary of *Included*** Trial Characteristics

|  | | **Lambrecht 2013** | **Raphael 2013** | **Sangthawan 2013** |
| --- | --- | --- | --- | --- |
| **Study characteristics** | | | | |
| Design | | Randomised controlled trial | Randomised double blind controlled trial | Randomised double blind controlled trial |
| Duration of follow-up | | Until OM < 3 | During OM period | 3 months |
| Location | | Belgium (single-center) | Netherlands (multi-center) | Thailand (SC) |
| **Participants** | | | | |
| Total number | | 58 | 34 | 139 |
| Age | | 30-78 years | 4-18 years | 18 years and above |
| Sex (% female) | | 72 | 44 | Quote: “most male” |
| Baseline characteristics | | Malignant neoplasms of the head and neck (all stages) and before undergoing chemotherapy and radiotherapy (1st cycle); intervention at treatment start and before the onset of oral mucositis (OM). | Haematological malignancies and before undergoing Hematopoietic stem cell transplantation (HSCT) and chemotherapy (first cycle) at the onset of OM. | Histologically documented diagnosis of head and neck cancer (all stages); Kanofsky performance status at least 70 and before undergoing radiation therapy (1st cycle) before the onset of OM. |
| OM status of area | | No prior history of OM. | No prior history of OM. | No prior history of OM. |
| OM status of participants | | No prior history of OM and mineral derivative treatment use. | No prior history of OM and mineral derivative treatment use. | No prior history of OM and mineral derivative treatment use. |
| Assessment of compliance | | Yes | Yes | Yes |
| **Intervention** | | | | |
| Intervention | | Caphosol® (Calcium phosphate) 1 minute rinse 15mL, 4 to 10 times daily plus standard care (Magic mouth wash, analgesics, antimycotics, antibiotics, parenteral tube) for 14 weeks (unspecified administration). | Caphosol® (Calcium phosphate; unspecified usage) plus standard care (unspecified) self-administered for the duration of OM. | Zinc sulfate oral syrup (5mg per 1cc) 10cc three times daily for 5 to 7 weeks self-administered. |
| Control | | Standard care (Magic mouth wash, analgesics, fluconazole, antibiotics, parenteral tube) for 14 weeks (unspecific administration). | NaCl 0.9% mouth rinse (unspecified usage) plus standard care (unspecified) self-administered for the duration of OM. | Placebo (unspecified components) three times daily for 5 to 7 weeks self-administered. |
| Other comparator/s | | None | None | None |
| **Outcome** | | | | |
| Primary | | OM graded NCT-CTCAE v3.0 scoring system on the incidence and duration of OM ≥ grade 3 by number of subjects and proportion. | OM graded NCI-CTCCAE v3.0 scoring system on the incidence and duration of OM > grade 1 (days). | OM graded NCT-CTC v2 scoring system on incidence and duration (weekly mean scores) grade 2 OM and pharyngitis. |
| Secondary | | Dysphagia, oral pain (Visual Analog Scale (VAS: 0-5); analgesic and opioid use measured in days. | Pain (age-appropriate pain scores); children reported product taste and mouth feeling appreciation daily) and tolerability (bad taste or intolerance) measured by analgesic use, blood culture, need for tube or parenteral feeding (days). | Oral and throat pain (VAS: 0-10); Treatment morbidity (IV fluids, body weight, hospitalisation); adverse effects of zinc sulfate. |
| Analysis method | | Parallel, Fisher exact test, for continuous: 2-tailed student’s *t*-test. | Chi-square or *t*-test for parametric scales. | *t*-test, chi-square or Fisher’s exact test. |
| Notes | | Funding source: “No funding was obtained. No financial relationships are applicable for this research.” | Funding source: study medications sponsored by EUSA Pharma (international division of Jazz pharmaceuticals) | Funding source: “Potential conflicts of interest-none.” |
| **Risk of bias** | | | | |
| Random sequence generation (selection bias) | Support for judgement | Quote: “Patients were randomized into two treatment arms”  Comment: Random component unspecified. | Quote: “Randomization was performed using an automatically generated list”  Comment: Automatically generated. Probably done. | Quote: “Block of four-randomization procedure … trial statistician generated the randomization sequence via a computerized random number generator”  Comment: Probably done. |
|  | Author’s judgement | Unclear risk | Low risk | Low risk |
| Allocation concealment (selection bias) | Support for judgement | Insufficient information to determine ‘yes’ or ‘no.’ | Quote: “…two patients stratified by the center and treatment (chemotherapy or HSCT)”; “coded prescriptions”  Comment: Central center and coded allocation. Probably done. | Quote: “In order to conceal the allocation process, a pharmacy staff was responsible for keeping the randomization list and assigned participants to the trial group.”  Comment: Probably done. |
|  | Author’s judgement | Unclear risk | Low risk | Low risk |
| Blinding (performance bias and detection bias) Patients | Support for judgement | No blinding and control group | Quote: “Double-blinded”  Comment: Probably done. | Quote: “Double-blind”  Comment: Probably done. |
|  | Author’s judgement | High risk | Low risk | Low risk |
| Blinding (performance bias and detection bias) Outcome assessors | Support for judgement | No blinding and control group. | Quote: “Double-blinded”  Comment: Probably done. | Quote: “Double-blind”  Comment: Probably done. |
|  | Author’s judgement | High risk | Low risk | Low risk |
| Incomplete outcome data (attrition bias) All outcomes | Support for judgement | Refused radiation therapy n= 1; declined to participate n = 1; treatment group transferred to control group n=4. Reasons unspecified. | n=1 patient excluded due to double registration; n=1 did not receive control rinse; Lost to follow up n=0; discontinued intervention n=0. | Drop-out reasons stated: n=1 died during course of radiotherapy; n=1 denied radiation therapy; n=1 left study due to personal reasons. |
|  | Author’s judgement | Unclear risk | Low risk | Low risk |
| Selective reporting (reporting bias) | Support for judgement | Allocation concealment missing; blinding and control omitted; random component unspecified. | None. | None. |
|  | Author’s judgement | High risk | Low risk | Low risk |
| Other bias | Support for judgement | Transferred non-compliant treatment group to control. | None. | None. |
|  | Author’s judgement | High risk | Low risk | Low risk |

|  | | **Jahangard-Rafsanjani 2013** | **Markiewicz 2012** | **Mansouri 2011** |
| --- | --- | --- | --- | --- |
| **Study characteristics** | | | | |
| Design | | Randomised controlled trial | Randomised double blind controlled trial | Randomised double blind controlled trial |
| Duration of follow-up | | 21 days and until OM resolved | Until granulocyte count was ≥ 0·2 g/L | 3 weeks |
| Location | | Iran (single-center) | Poland (single-center) | Iran (single-center) |
| **Participants** | | | | |
| Total number | | 77 | 40 | 60 |
| Age | | 18-55 years | 19-57 years | 15 years and above |
| Sex (% female) | | 43 | 40 | 33 |
| Baseline characteristics | | Acute myeloid leukemia (AML) or acute lymphocytic leukemia (ALL) undergoing allogenic HSCT (cycle unspecified); Karnofsky performance status ˂ 70%; Intervention at HSCT start and before the onset of OM. | AML or ALL or chronic myelogenous leukemia undergoing HSCT; intervention on the first day of conditioning before the onset of OM. | Hematologic malignancies undergoing high-dose chemotherapy conditioning regimen for allogenic HSCT (AML, ALL, CML, MDS) (cycle unspecified) intervention one day before conditioning and before the onset of OM. |
| OM status of area | | No description | No description | Blood culture before and after. |
| OM status of participants | | No description | No description | No description |
| Assessment of compliance | | Yes | None | Yes |
| **Intervention** | | | | |
| Intervention | | Selenium tablet (200mcg) twice daily during transplantation and 14 days after (staff administered) plus standard care (20 drops of nystatin every 3 hours, chewable sucralfate tablet 500mg every 8 hours and mouth washes containing chlorhexidine 0·02% plus 10cc diluted povidone iodine every 3 hours). | Calcium phosphate solution (equal volume, unspecified amount) four times daily self-administered until absolute neutrophil count ≥ 0·2 g/L. | Zinc sulfate capsule 220mg (50mg zinc elemental) twice daily 12-hour intervals for 3 weeks administered by hospital staff. |
| Control | | Placebo tablet (unspecified) twice daily during transplantation and 14 days after (staff administered) plus standard care. | Topical mouth care extract of salvia leaves twice daily, povidone-iodine mouth solution once daily, fluconazole mouth solution, glycerine (50mg), vitamin A (10g) and vitamin E (10g) with or without benzocoaine (2·5g) twice daily self-administered until absolute neutrophil count ≥ 0·2 g/L. | Placebo capsules (unspecified components) twice daily 12-hour intervals for 3 weeks administered by hospital staff. |
| Other comparator/s | | None | None | None |
| **Outcome** | | | | |
| Primary | | OM graded WHO toxicity scale (0-4) on the severity, incidence and duration OM > grade 2 in days; time to OM progression. | OM graded WHO toxicity scale (0-4) on the severity (mean), dysphagia (VAS: 0-5; mean) incidence (mean) and duration (days); pain (VAS: 0-10; mean); days to ANC > 0·5g/L; days to platelet count > 20g/L. | OM graded WHO (0-4) oral toxicity scale on the severity, duration, rate and start of OM. |
| Secondary | | Hematological indices – duration of ANC (Absolute Neutrophil Count) under 500 cells/mm³ (neutrophil, platelet engraftment and red cell culture). | Analgesics use (subjects/days); total Parenteral Nutrition (TPN: subjects/days); Granulocyte colony-stimulating factor (subjects); Acute graft-versus-host-disease (aGVHD-subjects); Degree of aGVHD; infectious complications (subjects). | Blood culture and weight comparison before and after treatment. |
| Analysis method | | Continuous variable and categorical data, chi-square, Fisher’s exact test. | Non-parametric: Mann-Whitney *U* tests; Fisher exact two-tailed tests; Yates chi-square tests. | Parallel; Mann-Whitney *U*;*t*-tests |
| Notes | | Funding source no description. | Funding source: EUSA Pharma supplied SCPR used in the trial; authors report no conflict of interest. | Funding source: Research grant Hematology-Oncology and SCT Research Center/Tehran University of Medical Sciences, Tehran, Iran. |
| **Risk of bias** | | | | |
| Random sequence generation (selection bias) | Support for judgement | Quote: “Patients were randomly allocated to selenium or control group in a blocked randomization schedule”  Comment: Probably not done. | Quote: “…randomized”  Comment: Random component not specified. | Quote: “We used randomized block in Microsoft Excel 2007 to randomize numbers”  Comment: Probably done. |
|  | Author’s judgement | High risk | Unclear risk | Low risk |
| Allocation concealment (selection bias) | Support for judgement | Quote: “Outcome assessor and the attending physician were blinded to patient’s allocation”  Comment: Probably done. | Insufficient information to determine ‘yes’ or ‘no.’ | Quote: “…opened the envelope containing codes”  Comment: Probably done. |
|  | Author’s judgement | Low risk | Unclear risk | Low risk |
| Blinding (performance bias and detection bias) Patients | Support for judgement | Quote: “Double-blind”  Comment: Probably done. | Quote: “…trial limitations include the impracticability of achieving double blinding with agents so different in appearance and in preadministration preparation…”  Comment: Probably not done. | Quote: “Double-blind”; “None of the participants and the staffs in the study were aware of the groups that the patients belonged”  Comment: Probably done. |
|  | Author’s judgement | Low risk | High risk | Low risk |
| Blinding (performance bias and detection bias) Outcome assessors | Support for judgement | Quote: “Double-blind”  Comment: Probably done. | Quote: “Nonblinded”  Comment: Probably not done. | Quote: “…one person unrelated to the study, who had the codes of drug and placebo”; “We followed all the patients every day through 3 weeks of study, without knowing the group of the patients”  Comment: Probably done. |
|  | Author’s judgement | Low risk | High risk | Low risk |
| Incomplete outcome data (attrition bias) All outcomes | Support for judgement | Discontinued intervention: n=1 patient died, n=2 patient non-adherent. Follow-up reasons unclear. | Insufficient information to determine ‘yes’ or ‘no.’ | Quote: “…all of them made it through the 3 weeks of trial”  Comment: Probably done. |
|  | Author’s judgement | Unclear risk | Unclear risk | Low risk |
| Selective reporting (reporting bias) | Support for judgement | Follow-up reasons unclear; funding source missing; OM characteristics undefined before commencing study; missing exclusion criteria; sequence generation unclear. | Purposeful non-blinding; attrition rate missing; OM characteristics undefined before commencing study; missing exclusion criteria. | OM characteristics undefined before commencing study; missing exclusion criteria. |
|  | Author’s judgement | High risk | High risk | High risk |
| Other bias | Support for judgement | The link between serum concentration levels and OM unclear: stratified sampling within treatment or control groups unspecified. | Control condition could be treatment condition. | Relevance of blood culture and weight comparisons to OM characteristics unclear. |
|  | Author’s judgement | High risk | High risk | High risk |

|  | | **Arbabi 2012** | **Mehdipour 2011** | **Buntzel 2010** |
| --- | --- | --- | --- | --- |
| **Study characteristics** | | | | |
| Design | | Randomised controlled trial | Randomised double blind controlled trial | Randomised double blind controlled trial |
| Duration of follow-up | | 20 weeks | 8 weeks | 7 years |
| Location | | Iran (single-center) | Iran (single-center) | Greece (multi-center: 6 centres) |
| **Participants** | | | | |
| Total number | | 50 | 45 | 39 |
| Age | | 18-79 years | 15 years and above | 38·7-83 years |
| Sex (% female) | | 48 | No description | 20 |
| Baseline characteristics | | Patients undergoing chemotherapy with same OM probability and Karnofsky performance ≥ 60 (1st cycle) intervention at treatment start and before the onset of OM. | Hematological malignancies and acute myeloid leukemia undergoing chemotherapy (cycle unspecified); intervention start unspecified; before the onset of OM. | Head and neck (squamous cell carcinoma) cancer undergoing chemoradiotherapy (cycle unspecified) intervention at 2 days before starting radiotherapy. |
| OM status of area | | No prior history of OM. | No prior history of OM. | No description. |
| OM status of participants | | No prior history of OM; salivary flows normal range. | No prior history of OM and mineral derivative treatment use. | No description of OM; decreased selenium levels. |
| Assessment of compliance | | No | Yes | No |
| **Intervention** | | | | |
| Intervention | | Zinc sulfate capsule 220mg three times daily self-administered until the end of chemotherapy. | Zinc sulfate mouthwash (0·2% dilution) rinse twice daily for 14 days administered by an investigator. | Sodium selenite oral fluid 500µg 1-hour before radiotherapy; 300µg during weekends and official holidays for unspecified length; unspecified administration. |
| Control | | Placebo capsule (similar shape, taste, color to intervention) three times daily self-administered until the end of chemotherapy. | Not specified. | Not specified. |
| Other comparator/s | | None | Chlorhexidine gluconate mouthwash (0·2% dilution) rinse twice daily for 14 days administered by an investigator. | None |
| **Outcome** | | | | |
| Primary | | OM graded WHO (0-4) oral toxicity scale; xerostomia and pain intensity (VAS: 0-10; weekly mean). | OM graded Spijkevet scale (0-4) on severity (length of OM lesions). | OM weekly mean graded RTOG (Radiation Therapy Oncology Group-0-4) scale on xerostomia (dry mouth), stomatitis (inflammation of oral mucosa), ageusia (loss of taste) and dysphagia (problems in nutrition) and selenium serum concentration effects. |
| Secondary | | OM and xerostomia recovery (weekly mean); time effect on OM, xerostomia and pain intensity (Friedman); QoL (EORTC QLQ-OES18). | None. | Pain and analgesic use measured in days. |
| Analysis method | | Independent *t*-tests; Mann-Whitney *U* test, Friedman test | ANOVA; Independent *t*-tests. | Fisher’s exact test; student’s *t*-test. |
| Notes | | Funding source no description. | Funding source: Supported by the vice chancellor for research, Tabriz University of Medical Sciences. | Funding source: biosyn Arzneimittel GmbH, Fellbach, Germany. |
| **Risk of bias** | | | | |
| Random sequence generation (selection bias) | Support for judgement | Quote: “Patients were block randomized into two groups”  Comment: Probably not done. | Quote: “…mouthwashes were coded ‘A’ and ‘B’ letters on the bottles …and were administered to the groups by a simple random method”  Comment: Probably done. | Quote: “Randomisation was performed and the patient received information about the treatment arm”  Comment: Random component unspecified. |
|  | Author’s judgement | High risk | Low risk | High risk |
| Allocation concealment (selection bias) | Support for judgement | Quote: “…zinc sulfate drug prescriptions were carried out by patient’s own oncologists.”  Comment: Probably not done. | The mouthwashes were coded “A” and “B” letters on the bottles by the Faculty of Pharmacy blinded to investigators. | Insufficient information to determine ‘yes’ or ‘no.’ |
|  | Author’s judgement | High risk | Low risk | Unclear risk |
| Blinding (performance bias and detection bias) Patients | Support for judgement | Quote: “Double-blind”  Comment: Probably done. | Quote: “…subjects were blinded to the type of the mouthwash”  Comment: Probably done. | Insufficient information to determine ‘yes’ or ‘no.’ |
|  | Author’s judgement | Low risk | Low risk | Unclear risk |
| Blinding (performance bias and detection bias) Outcome assessors | Support for judgement | Quote: “Double-blind”; “The student and specialist were blinded to the randomization and treatment.”  Comment: Probably done. | Quote: “Investigators as well as the subjects were blinded to the type of mouthwash”  Comment: Probably done. | Insufficient information to determine ‘yes’ or ‘no.’ |
|  | Author’s judgement | Low risk | Low risk | Unclear risk |
| Incomplete outcome data (attrition bias) All outcomes | Support for judgement | Insufficient information to determine ‘yes’ or ‘no’ | Quote: “…all 30 patients completed the course of study”  Comment: Probably done | Quote: “After 7 years of recruitment, blood samples of 113 patients were measured and only 39 out of 93 patients with selenium deficiency decided to take part”  Comment: Probably not done. |
|  | Author’s judgement | Unclear risk | Low risk | High risk |
| Selective reporting (reporting bias) | Support for judgement | Attrition rate missing; funding source missing. | No control data; results presented as graph difficult to follow and data not reported in text. | Allocation concealment, blinding and attrition rate missing; OM characteristics undefined before commencing study; results graph difficult to follow with no data reference in text. |
|  | Author’s judgement | High risk | High risk | High risk |
| Other bias | Support for judgement | None | Control condition could be treatment condition. | Quote: “The individual patient file was transferred to the sponsor who was responsible for monitoring.”  Comment: Conflict of interest.  Relevance of blood culture to OM characteristics unclear. |
|  | Author’s judgement | Low risk | High risk | High risk |

|  | | **Lin 2010a** | **Watanabe 2010** | **Madan 2008** |
| --- | --- | --- | --- | --- |
| **Study characteristics** | | | | |
| Design | | Randomised controlled trial | Randomised double blind controlled trial | Randomised double blind controlled trial |
| Duration of follow-up | | 12 months | 10 months | 6 weeks |
| Location | | Taiwan | Japan (single-center) | India (single center) |
| **Participants** | | | | |
| Total number | | 97 | 31 | 80 |
| Age | | 36·47-64·77 years | 35-78 years | 18 years and above |
| Sex (% female) | | 20 | 23 | 17 |
| Baseline characteristics | | Head and neck (nasopharangeal carcinoma) cancer or oral cancer (all stages) undergoing radiotherapy (recurrent or not); intervention start unspecified. | HNC (stage II-IV) undergoing radiotherapy or radiochemotherapy (cycle unspecified) intervention at treatment start. | Head and neck malignancies (stage II -IV) undergoing radiotherapy (1^st^ cycle) intervention at treatment start before the onset of OM. |
| OM status of area | | No description. | No description; blood culture. | No prior history of OM and mineral derivative treatment use. |
| OM status of participants | | No description. | No description. | No prior history of OM and mineral derivative treatment use. |
| Assessment of compliance | | Yes | No | Yes |
| **Intervention** | | | | |
| Intervention | | Oral zinc capsules (25 mg Pro-Z) 3 times daily for 2 months; unspecified administration. | Polaprezinc (Promac granules®15%) 0.5g dissolved in 20ml of 5% sodium alginate solution 4 times daily (3min oral rinse then swallow) until end of radiotherapy; unspecified administration. | 1% Povidone-iodine 10ml mouthwash, twice daily for 6 weeks self-administered. |
| Control | | Soybean oil capsules 3 times daily for 2 months; unspecified administration. | Azulene (Azunol® Gargle liquid 4%) in 100ml water 4 times daily (3min oral rinse no swallow) until end of radiotherapy; unspecified administration. | Plain water 10ml mouthwash, twice daily for 6 weeks self-administered. |
| Other comparator/s | | None | None | Chlorhexidine (0·12%); salt soda bicarbonate. |
| **Outcome** | | | | |
| Primary | | OM bi-weekly group proportion survival times graded (2 or 3) Acute Radiation Morbidity Scoring Criteria (0-4) on duration, severity and zinc serum levels. | OM, xerostomia and taste disturbances graded CTCAE version 3.0 scoring system on incidence, severity (patient number and proportion). | OM graded WHO (0-4) oral toxicity scale on severityand onset mean weekly scores. |
| Secondary | | OM mean range between 1 and 2 weeks after radiotherapy completion compared to week 8 of treatment (treatment group versus control). | Relative Risk measures on analgesic frequency and disability to oral intake on OM, pain, xerostomia, taste disturbances. | None. |
| Analysis method | | Chi-square, Fisher exact tests, student *t*-test, Kaplan-Meier survival method, log ranks. | Parametric: *t*-tests  Non-parametric: Mann-Whitney *U-*test, Fisher’s exact probability test, Relative Risk and Confidence Intervals. | ANOVA, and chi-square test. |
| Notes | | Funding source no description. | Funding source no description. | Funding source: Manipal University, India. |
| **Risk of bias** | | | | |
| Random sequence generation (selection bias) | Support for judgement | Quote: “The RV.UNIFORM (0, 1) function in SPSS for Windows (SPSS, Inc., Chicago, IL) was adopted to generate random numbers…”  Comment: Probably done. | Quote: “Randomly assigned”  Comment: Random component unspecified. | Quote: “The mouthwashes were numbered randomly from 1 to 80 by the mouthwash manufacturer (Dispensing Wing, KMC Pharmacy, Manipal, India).”  Comment: Probably not done. |
|  | Author’s judgement | Low risk | Unclear risk | High risk |
| Allocation concealment (selection bias) | Support for judgement | Quote: “…assign distinct random permuted blocks to patients.”  Comment: Probably done. | Insufficient information to determine ‘yes’ or ‘no.’ | A patient assigned a particular number was given the mouthwash with the same number. Probably not done. |
|  | Author’s judgement | Low risk | Unclear risk | High risk |
| Blinding (performance bias and detection bias) Patients | Support for judgement | Quote: “Double-blind”  Comment: Probably done.. | Insufficient information to determine ‘yes’ or ‘no.’ | Quote: “Double-blind”  Comment: Probably done. |
|  | Author’s judgement | Low risk | Unclear risk | Low risk |
| Blinding (performance bias and detection bias) Outcome assessors | Support for judgement | Quote: “The observer was blind to the treatment vs. control groups to eliminate any systematic bias in assessing treatment effects.”  Comment: Probably done. | Insufficient information to determine ‘yes’ or ‘no.’ | Quote: “It was revealed to the investigator only at the end of the study.”; “Double-blind.”  Comment: Probably done. |
|  | Author’s judgement | Low risk | Unclear risk | Low risk |
| Incomplete outcome data (attrition bias) All outcomes | Support for judgement | Patient decided to receive herbal treatment (n=1); Patient decided to receive alternative treatment with herb drug (n=1). | Insufficient information to determine ‘yes’ or ‘no.’ | Death due to tumor-related complications (n=8). |
|  | Author’s judgement | Low risk | Unclear risk | Low risk |
| Selective reporting (reporting bias) | Support for judgement | OM characteristics undefined before commencing study; exclusion characteristics missing; funding source missing; cumulative weekly data without the number of patients. | Attrition rate, blinding and randomization missing; OM characteristics undefined before commencing study; exclusion criteria missing. | Uncertain if manufacturer followed due protocol such as randomisation. |
|  | Author’s judgement | High risk | High risk | High risk |
| Other bias | Support for judgement | Soybean as a non-active control group was not justified; did not match serum zinc levels directly to OM condition in the method and results: stratified sampling within treatment or control groups unclear. | None. | Quote: “Patient compliance was assessed by weekly checking of the level of mouthwash left in the bottle.”  Comment: unblinding. |
|  | Author’s judgement | High risk | Low risk | High risk |

|  | | **Lin 2006b** | **Vokurka 2005** | **Ertekin 2004** |
| --- | --- | --- | --- | --- |
| **Study characteristics** | | | | |
| Design | | Randomised controlled trial | Randomised double blind controlled trial | Randomised double blind controlled trial |
| Duration of follow-up | | 12 months | January 2002-June 2004 | 13 weeks |
| Location | | Taiwan (single center) | Czech Republic (multi-center) | Turkey (single center) |
| **Participants** | | | | |
| Total number | | 100 | 148 | 27 |
| Age | | 39-62 years | 20-70 years | 18-71 years |
| Sex (% female) | | 14 | 39 | 22 |
| Baseline characteristics | | Head and neck cancer (stage I-IV) undergoing chemoradiotherapy (cycle unspecified) from first to last day of radiotherapy; OM status unspecified. | Patients undergoing high dose chemotherapy and autologous peripheral stem cell transplantation (unspecified cycle); intervention start unspecified. | Head and neck cancer undergoing radiotherapy; (unspecified cycle); Karnofsky’s performance status ≥ 70; intervention at treatment start. |
| OM status of area | | Pre-treatment serum zinc levels assessed. | OM incidence (WHO grades 0-4) characterised at intervention during treatment. | No prior history of OM and mineral derivative treatment use. |
| OM status of participants | | No description. | No prior history of OM and radiotherapy treatment. | No prior history of OM and mineral derivative treatment use. |
| Assessment of compliance | | Yes | No | Yes |
| **Intervention** | | | | |
| Intervention | | Zinc capsules (25 mg Pro-Z) 3 times daily for 2 months; unspecified administration. | Povidone-iodine solution diluted 1:100 (Betadine 1ml and 100 ml water for injection) mouth wash 4 times 2 minute gargle daily administered by study nurse for unspecified completion length. | Zinc sulfate (50mg Zinc; Zinco 220 capsule) 3 times daily at 8-hour intervals during radiotherapy and 6 weeks after treatment; unspecified administration. |
| Control | | Soybean oil capsules 3 times daily for 2 months; unspecified administration. | Saline (NaCl 9% water solution) mouth wash 4 times 2 minute gargle daily administered by study nurse for unspecified completion length. | Empty placebo capsules taken 3 times daily at 8-hour intervals during radiotherapy and 6 weeks after treatment; unspecified administration. |
| Other comparator/s | | No | No | No |
| **Outcome** | | | | |
| Primary | | OM bi-weekly group proportion survival times graded (2 or 3) Acute Radiation Morbidity Scoring Criteria (0-4) on duration and severity. | OM graded WHO (0-4) oral toxicity scale on incidence, cumulative incidence, severityand duration on the proportion of grade types; Oral pain (VAS: 0-10; analgesics use); Mouthwash tolerability (VAS: 0-5). | OM graded RTOG on severity (grade), onset (week) and radiotherapy dose OM developed. |
| Secondary | | OM mean range after radiotherapy between 1 week and 2 weeks after treatment completion compared to last week of treatment (treatment group versus control); Side effects of Zinc in blood culture. | Infectious complications. | None. |
| Analysis method | | Chi-square, Fisher exact tests, student *t*-test, Kaplan-Meier survival method. | Mann-Whitney *U* test and Fisher’s exact test. | Mann-Whitney *U* test, Fisher’s exact test, chi-square test, Friedman variation analysis. |
| Notes | | Funding source: Chi-Mei Foundation Medical Centre (CMFHR9201). | Funding source no description. | Funding source no description. |
| **Risk of bias** | | | | |
| Random sequence generation (selection bias) | Support for judgement | Quote: “RV.UNIFORM (0, 1) function in SPSS for Windows (SPSS, Inc., Chicago, IL) to generate random numbers…”  Comment: Probably done. | Quote: “Patients randomized to study group A… group B.”  Comment: Random component unspecified. | Quote: “…patients were randomly assigned…”  Comment: Random component unspecified. |
|  | Author’s judgement | Low risk | Unclear risk | Unclear risk |
| Allocation concealment (selection bias) | Support for judgement | Quote: “The drug contents were not revealed, even to the principal investigator, until the end of the experiment.”  Comment: Probably done. | Insufficient information to determine ‘yes’ or ‘no.’ | Insufficient information to determine ‘yes’ or ‘no.’ |
|  | Author’s judgement | Low risk | Unclear risk | Unclear risk |
| Blinding (performance bias and detection bias) Patients | Support for judgement | Quote: “Double-blind”  Comment: Probably done. | Quote: “The solutions were freshly prepared every morning and their composition was blinded to the patients.”  Comment: Probably done. | Insufficient information to determine ‘yes’ or ‘no.’ |
|  | Author’s judgement | Low risk | Low risk | Unclear risk |
| Blinding (performance bias and detection bias) Outcome assessors | Support for judgement | Quote: “Double-blind”  Comment: Probably done. | Insufficient information to determine ‘yes’ or ‘no.’ | Insufficient information to determine ‘yes’ or ‘no.’ |
|  | Author’s judgement | Low risk | Unclear risk | Unclear risk |
| Incomplete outcome data (attrition bias) All outcomes | Support for judgement | n=2 dropped out but reasons unstated. | Insufficient information to determine ‘yes’ or ‘no.’ | n=1 patients died; n=1 withdrew from study; n=1 did not attend the 6-week control visit after treatment. Reasons for withdrawal and nonattendance unspecified. |
|  | Author’s judgement | Unclear risk | Unclear risk | Unclear risk |
| Selective reporting (reporting bias) | Support for judgement | Attrition reasons unclear. | Allocation concealment and attrition rates missing; blinding and random component unclear; funding source missing. | Allocation concealment and blinding missing; random method and attrition rate reasons unclear; funding source missing. |
|  | Author’s judgement | High risk | High risk | High risk |
| Other bias | Support for judgement | Soybean as non-active control group was not justified; zinc side effects did not directly relate to OM condition in the method and results. Suggested a connection from “wound healing and metabolic response” but did not show numerical a priori in the analysis. | Does not distinguish pain scores between treatment and control. | None. |
|  | Author’s judgement | High risk | High risk | Low risk |

|  | | **Papas 2003** |
| --- | --- | --- |
| **Study characteristics** | | |
| Design | | Randomised double blind controlled trial |
| Duration of follow-up | | Not specified |
| Location | | United States (single center) |
| **Participants** | | |
| Total number | | 95 |
| Age | | 18-70 years |
| Sex (% female) | | 6 |
| Baseline characteristics | | HSCT (AML or ALL or chronic myelogenous leukemia, Hodgkin’s disease, non-Hodgkin’s lymphoma, multiple myelomas, myelodysplastic syndrome, breast cancer, ovarian cancer, other) (cycle unspecified); intervention start one week prior to treatment at screening. |
| OM status of area | | No description. |
| OM status of participants | | No description. |
| Assessment of compliance | | Yes |
| **Intervention** | | |
| Intervention | | Caphosol® (Calcium phosphate) rinse 4 times daily, 10 times daily when OM developed until engraftment and the resolution of OM; prior to HSCT 4 topical fluoride treatments of 1%F as neutral 2% NaF gel at screening; administered by trained unit nurses. |
| Control | | Aqueous sodium fluoride 0·01% 30ml rinse 4 times daily, 10 times daily when OM developed until engraftment and the resolution of OM; prior to HSCT 4 topical treatments with placebo gel administered by trained unit nurses. |
| Other comparator/s | | No |
| **Outcome** | | |
| Primary | | OM graded National Institute of Dental and Craniofacial Research (NIDCR: 0-5) scoring on duration (days > 1) and peak OM; days to ANC (onset of neutrophil engraftment); daily log (assisted by nurse of study dentist). |
| Secondary | | Pain (VAS: 0-100); pain duration and peak (days); self-administered morphine (days of morphine); days of post-BMT (hospital stay post-stem-cell infusion); days of fever. |
| Analysis methods | | ANOVA F; Mann-Whitney *U* test. |
| Notes | | Funding source no description. |
| **Risk of bias** | | |
| Random sequence generation (selection bias) | Support for judgement | Quote: “…patients were randomized…”  Comment: Random component unspecified. |
|  | Author’s judgement | Unclear risk. |
| Allocation concealment (selection bias) | Support for judgement | Insufficient information to determine ‘yes’ or ‘no.’ |
|  | Author’s judgement | Unclear risk |

| Blinding (performance bias and detection bias) Patients | Support for judgement | Quote: “double-blind”  Comment: Probably done. |
| --- | --- | --- |
|  | Author’s judgement | Low risk |
| Blinding (performance bias and detection bias) Outcome assessors | Support for judgement | Quote: “double-blind”  Comment: Probably done. |
|  | Author’s judgement | Low risk |
| Incomplete outcome data (attrition bias) All outcomes | Support for judgement | n=1 refused rinse; n=1 too ill to participate.  Quote: ‘…we attribute the high level of compliance to the diligence of the nursing staff…’  Comment: Compliance reasons stated. |
|  | Author’s judgement | Low risk |
| Selective reporting (reporting bias) | Support for judgement | Allocation concealment missing; blinding and random method unclear; OM characteristics and exclusion criteria missing; funding source missing. |
|  | Author’s judgement | High risk |
| Other bias | Support for judgement | None. |
|  | Author’s judgement | Low risk |

**Table 2: Characteristics of excluded studies (n=9)**

| **Study** | **Reason for exclusion** |
| --- | --- |
| Pettit, 2013^47^ | Prospective audit. Not randomised. |
| Stokman, 2012^46^ | No control group and not randomised. |
| Waśko-Grabowska, 2011^48^ | Not randomised. Control group treated before treatment group. |
| Bodnar, 2008^49^ | Disease listed as side effect and not part of the primary study. |
| Vokurka, 2006^50^ | Supplementary material to a previously conducted study. |
| Nagy, 2000^52^ | Administered vitamin (K1) supplements. |
| Buntzel, 2010b^54^ | Same study, different title and journal. |
| Ertekin, 2003b^51^ | Could not abstract data from positive and negative streptococcus results. |
| Meca, 2009 | Could not abstract data from streptococcus results |
